# On the Jahn–Teller Effect in Silver Complexes of Dimethyl Amino Phenyl Substituted Phthalocyanine †

**DOI:** 10.3390/molecules28207019

**Published:** 2023-10-10

**Authors:** Martin Breza

**Affiliations:** Department of Physical Chemistry, Slovak Technical University, Radlinskeho 9, SK-81237 Bratislava, Slovakia; martin.breza@stuba.sk

**Keywords:** DFT, TD-DFT, symmetry descent, electron states, vibronic interactions

## Abstract

The structures of Ag complexes with dimethyl amino phenyl substituted phthalocyanine ^m^[dmaphPcAg]^q^ of various charges q and in the two lowest spin states m were optimized using the B3LYP method within the D_4h_ symmetry group and its subgroups. The most stable reaction intermediate in the supposed photoinitiation reaction is ^3^[dmaphPcAg]^−^. Group-theoretical analysis of the optimized structures and of their electron states reveals two symmetry-descent mechanisms. The stable structures of maximal symmetry of complexes ^1^[dmaphPcAg]^+^, ^3^[dmaphPcAg]^+^, ^2^[dmaphPcAg]^0^, and ^4^[dmaphPcAg]^2−^ correspond to the D_4_ group as a consequence of the pseudo-Jahn–Teller effect within unstable D_4h_ structure. Complexes ^4^[dmaphPcAg]^0^, ^1^[dmaphPcAg]^−^, ^3^[dmaphPcAg]^−^, and ^2^[dmaphPcAg]^2−^ with double degenerate electron ground states in D_4h_ symmetry structures undergo a symmetry descent to stable structures corresponding to maximal D_2_ symmetry, not because of a simple Jahn–Teller effect but due to a hidden pseudo-Jahn–Teller effect (strong vibronic interaction between excited electron states). The reduction of the neutral photoinitiator causes symmetry descent to its anionic intermediate because of vibronic interactions that must significantly affect the polymerization reactions.

## 1. Introduction

**Figure 1:**
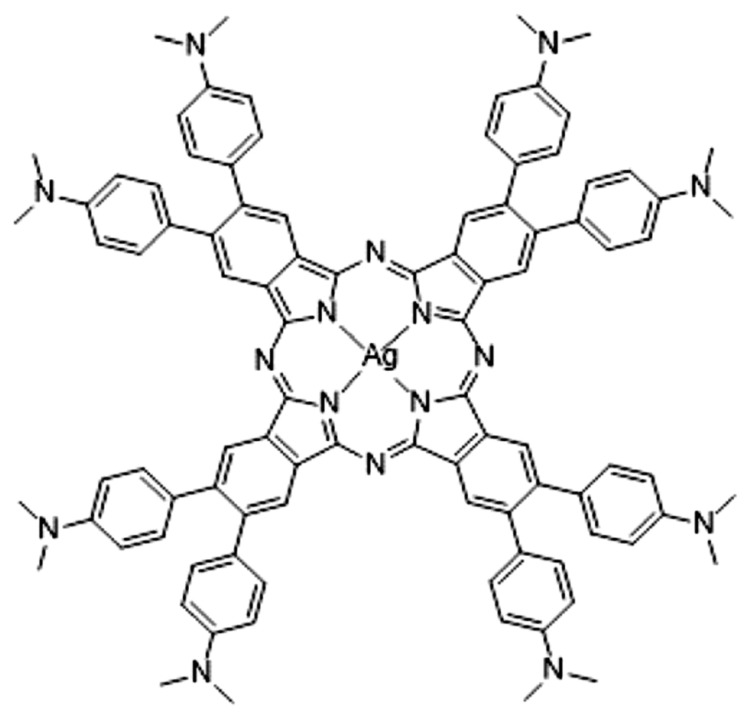
Structure of ^2^[dmaphPcAg]^0^ [1].

New photosensitive systems with improved efficiency for initiating free-radical and/or cationic polymerizations under light activation are developed using sophisticated chemical treatments. Very recently, Breloy et al. [1] synthesized dimethyl amino substituted phthalocyanine dmaphPcH_2_ and its Ag(II) complex ^2^[dmaphPcAg]^0^ (Figure 1). Photoexcitation of [dmaphPcAg]^0^ in the presence and absence of an iodonium salt produced acidic and radical species that initiated cationic and free-radical polymerizations, respectively. The photoinitiator properties were investigated via spectroscopic and quantum-chemical methods. The polymerization kinetics in laminate and under air was studied. In the first step, irradiation (385 and 405 nm) caused a reduction of Ag(II) to Ag(I), and nitrogen-centered radicals were formed. Subsequently, Ag nanoparticles and carbon-centered radicals developed. Intermediates [dmaphPc]^−^, Ag^0^ nanoparticles, and [dmaphPcAg]^q^ complexes, q = −1 → +1, are supposed within the proposed reaction mechanism. DFT calculations indicate a D_4_ → D_2_ symmetry descent in [dmaphPcAg]^q^ complexes which could be ascribed to the Jahn–Teller (JT) effect. The aim of our recent study is to shed more light on this problem and to verify the above explanation using a group-theoretical analysis of the DFT-optimized structures of [dmaphPcAg]^q^ complexes within the highest possible D_4h_ symmetry group and its subgroups.

## 2. Theoretical Background

According to the Jahn–Teller theorem [2], any nonlinear configuration of atomic nuclei in a degenerate electron state is unstable. Hence, there is at least one such configuration of lower symmetry where the above degeneracy is removed. In other words, the multidimensional representation in the high-symmetric (HS) structure is split into nondegenerate representations in the low-symmetric (LS) structure.

The JT active coordinate *Q*_JT_ describes the abovementioned symmetry descent. If the potential energy surface *E* = f(*Q*_i_) of N atoms is a function of 3N − 6 independent nuclear coordinates *Q*_i_, for the JT ‘unstable’ HS structure, the following relation for any *Q*_JT_ is valid:

(1) $${\left(\frac{\partial E}{\partial Q_{\mathrm{JT}}}\right)}_{\mathrm{HS}}\neq 0$$

The HS → LS geometry change described by *Q*_JT_ is connected with an energy decrease which is denoted as the JT stabilization energy *E*_JT:_

*E*_JT_ = *E*_HS_ − *E*_LS_ (2)

An analogous symmetry descent and energy decrease for pseudodegenerate electron states is known as the pseudo-Jahn–Teller (PJT) effect. It can be observed for a sufficiently strong vibronic interaction and relatively small energy difference Δ_ij_ between the interacting electron states Ψ_i_ and Ψ_j_ [3].

In some cases, the existence of LS structures cannot be explained by the JT effect of the degenerate ground electron state nor by the PJT interactions of the non-degenerate ground electron state with low-lying excited states in the HS structure (e.g., because of their different spin multiplicity). (P)JT effects in these cases are “hidden” in the excited states, which can undergo vibronic interactions as well. The vibronic interaction within the degenerate excited state or between two (or more) excited states of suitable symmetries can be so strong that the lower energy surface penetrates the potential surface of the ground electron state corresponding the HS structure and becomes the lowest state in the LS structure (i.e., its ground electron state). These consequences of the strong JT and PJT vibronic interactions within excited electron states are known as the hidden JT (HJT) and hidden PJT (HPJT) effects, respectively [4].

(P)JT potential surfaces can be described using an analytical function based on perturbation theory. In the simplest case of single distortion coordinate *Q* and double electron degeneracy or two interacting electron states of different symmetries, we obtain their potential energy surface *E*(*Q*) in the form

(3) $$E\left(Q\right)=\frac{1}{2}KQ^{2}\pm [\frac{{∆}_{\mathrm{ij}}^{2}}{4}+F^{2}Q^{2}{]}^{1/2}$$

where Δ_ij_ = 0 for double electron degeneracy (JT effect) or Δ_ij_ > 0 is the energy difference between both interacting electron states (PJT effect) in the undistorted geometry, *K* is the primary force constant (without vibronic coupling), and *F* is the vibronic coupling constant [3]. However, the search for their extremal points corresponding to the ‘stable’ or ‘unstable’ (P)JT structures is too complicated for large molecular systems. In such cases, a group-theoretical treatment must be used.

The epikernel principle method [5,6] is based on the *Q*_JT_ symmetry in the HS structure. A nonzero value of the integral

(4) $$<\Psi_{i}\left|\frac{\partial^{}\hat{H}}{\partial Q_{\mathrm{JT}}}\right|\Psi_{j}>\neq 0$$

for the interacting electron states Ψ_i_ and Ψ_j_ and the full-symmetric Hamilton operator $\hat{H}$ demands that the direct product

Γ_i_* ⊗ Λ_JT_ ⊗ Γ_j_ (5)

where Γ_i_, Λ_JT_, and Γ_j_ are representations of Ψ_i_, *Q*_JT_, and Ψ_j_, respectively (the asterisk denotes a complex conjugate value), must contain a full-symmetric representation. Alternatively:

Λ_JT_ ∈ Γ_i_* ⊗ Γ_j_ (6)

In the case of the JT effect and the degenerate states Ψ_i_ = Ψ_j_, i.e., Γ_i_ = Γ_j_, we obtain the even stronger condition

Λ_JT_ ∈ [Γ_i_ ⊗ Γ_i_]^+^ (7)

where […]^+^ denotes the symmetric direct product.

The epikernel principle states that the extrema of a JT energy surface correspond to the kernel K(G, Λ_JT_) or epikernel E(G, Λ_JT_) subgroups of the HS parent group G. Kernels contain the symmetry operations of G that leave the Λ_JT_ representation invariant. The symmetry operations of epikernels leave invariant only some components of the degenerate Λ_JT_ representation.

The epikernel principle was originally formulated for the systems in degenerate electron states only [5,6], but it has been successfully extended (except Equation (7)) to pseudodegenerate electron states as well [7]. Its drawback is in the restriction to JT active coordinates that have been derived within perturbation theory for the linear Taylor expansion of the perturbation only. This method might offer incomplete results in some cases (e.g., in systems with C_5_ rotations) [7].

The method of step-by-step symmetry descent [8,9] is based on consecutive splitting of a degenerate electron state within a JT symmetry descent. The vibronic interaction causes instability of the HS structure, and thus, in the sense of the JT theorem [2], some symmetry elements are removed. The probability of the symmetry elements removal decreases with the number of these elements. It implies that the symmetry decrease to the immediate subgroups with the lowest number of the removed symmetry elements are more probable than to other subgroups. The multidimensional representation corresponding to the degenerate electron state can be split within symmetry descent to an immediate subgroup of the parent HS group G (see Table S1 in Supplementary Materials). If the structure corresponding to this subgroup is in a nondegenerate electron state, it is JT stable and the symmetry descent stops. If the structure corresponding to an immediate subgroup of G is in a degenerate electron state described by a multidimensional representation (a JT unstable structure), the symmetry descent continues to its immediate subgroups and the whole procedure is repeated. As every group can have several immediate subgroups, various symmetry descent paths are possible and the JT stable structures can correspond to various LS symmetry groups. The only condition is its nondegenerate electron state (i.e., one-dimensional representation), obtained through splitting the degenerate electron state (i.e., multidimensional representation) of the HS structure of the parent group G.

## 3. Results

**Figure 2:**
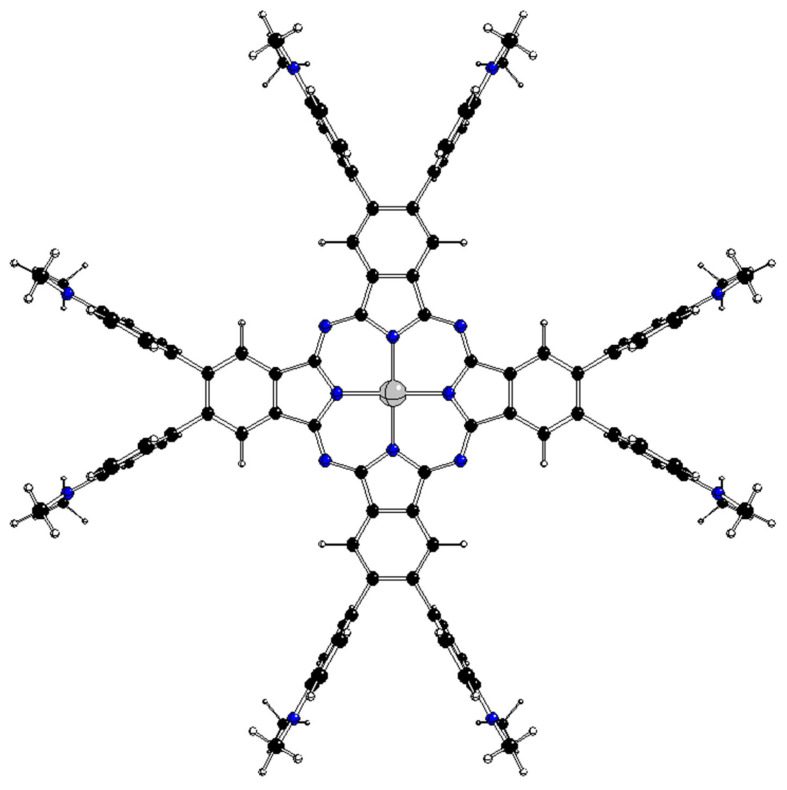
Optimized D_4h_ structure of ^2^[dmaphPcAg]^0^ (C—black, N—blue, H—white, Ag—grey).

The highest possible structures of [dmaphPcAg]^q^ complexes are of the D_4h_ symmetry group with side phenyl groups perpendicular to the central phthalocyanine plane (Figure 2). Because of the great number of possible mutual orientations of dimethyl amino phenyl groups in less symmetric structures, we restricted our study to the stable structures of the maximal symmetry group only. Another restriction is implied by the inability of standard DFT methods to optimize the atomic configurations in degenerate electron states [10]. The results of the geometry optimizations of the ^m^[dmaphPcAg]^q^ complexes with total charges q = +1 → −2 in the two lowest spin states m are presented in Table 1. It is interesting that the [dmaphPcAg]^q^ complexes in the triplet spin state are more stable than these ones with the same charge in the singlet spin state. ^3^[dmaphPcAg]^−^ of D_2_ symmetry is the most stable complex under study. It must be mentioned that the use of an unrestricted ‘broken symmetry’ treatment [11] results in zero spin populations and does not reduce the energy of the systems studied. Therefore, only the restricted Kohn–Sham formalism has been used in subsequent TD-DFT calculations of our complexes in singlet ground states.

Further inspection of Table 1 shows that the studied complexes can be divided into two categories:

(i) Category I contains complexes ^1^[dmaphPcAg]^+^, ^3^[dmaphPcAg]^+^, ^2^[dmaphPcAg]^0^, and ^4^[dmaphPcAg]^2−^ with optimized structures of D_4h_ (unstable) and D_4_ (stable) symmetry groups.
(ii) Category II contains complexes ^4^[dmaphPcAg]^0^, ^1^[dmaphPcAg]^−^, ^3^[dmaphPcAg]^−^, and ^2^[dmaphPcAg]^2−^, where only their stable optimized structures of the D_2_ symmetry group were found, while the D_4h_ optimized structures were absent. This can be explained by the electron configurations of the D_4h_ complexes in Table 2. We may conclude that the category II complexes should contain partially occupied e_g_ molecular orbitals, which implies E_g_ or E_u_ ground electron states. Hence, they are not accessible through standard DFT methods.

### 3.1. Category I Complexes

**Figure 3:**
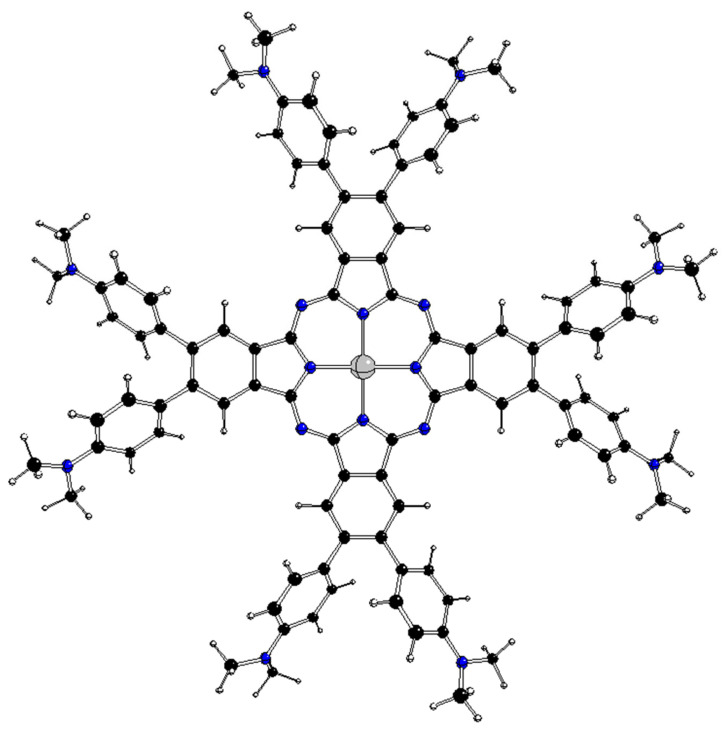
Optimized D_4_ structure of ^2^[dmaphPcAg]^0^ (see Figure 2 for atom notation).

The optimized D_4h_ structures of these complexes are unstable due to several imaginary vibrations of Λ_im_ representations (Table 1) that coincide with the JT active coordinates. The JT stabilization energies E_JT_ correspond to D_4h_ → D_4_ symmetry decrease. Stable D_4_ structures with equally rotated phenyl rings (Figure 3) and without any imaginary vibration correspond to the K(D_4h_, Λ_im_) kernel subgroups for the coordinate of the a_1u_ representation. As indicated by its wavenumber ν_im_, the energies of the corresponding vibrations are comparable with the highest energy ones in all category I complexes. The optimized structures of D_2d_ and C_4v_ symmetries are not stable (not presented), and hence only the D_4_ ones fulfill the condition of the stable structure of the highest symmetry group.

For known ground state Γ_0_ and JT coordinate Λ_im_ representations, the Equations (4) and (5) can be used to determine the excited-state representations Γ_exc_ (see Table 1) interacting with the ground electron state in D_4h_ structures of our complexes. The comparison of the TD-DFT calculated electron states in the corresponding D_4h_ and D_4_ structures (Table 3) shows that the energy difference *E*_exc_ between the PJT interacting states increases after symmetry descent as a consequence of their vibronic interaction. This confirms the correct assignment of the corresponding states in both groups because the standard treatment based on similarity of their oscillator strengths *f* is hardly usable in our cases. The ground states in the D_4h_ structures also correspond to those in their D_4_ subgroups.

We can see (Table 3) that the PJT active excited states in the D_4h_ structures are relatively high. This indicates that their excitation energies are less important for possible vibronic interactions than the energies of JT active coordinates.

### 3.2. Category II Complexes

**Figure 4:**
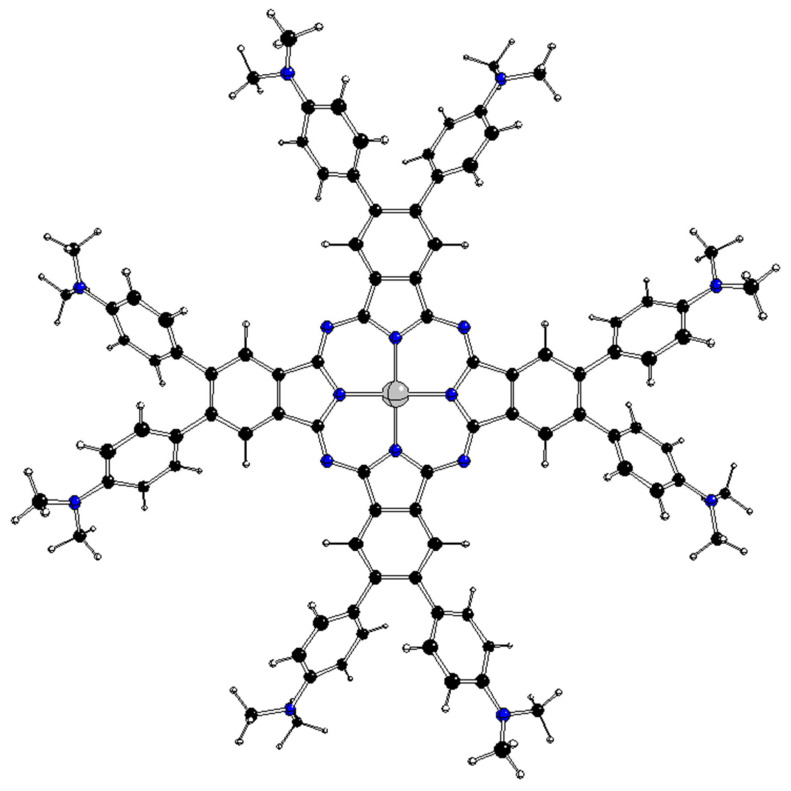
Optimized D_2_ structure of ^1^[dmaphPcAg]^−^ (see Figure 2 for atom notation).

As mentioned above, the ground state of the D_4h_ structures of these complexes is double degenerate (E_g_ or E_u_ representations), and only the stable D_2_ structures (Figure 4) were obtained through geometry optimizations. Possible representations of the corresponding JT active coordinates can be obtained via the symmetric direct products for the HS group:

[E_g_ ⊗ E_g_]^+^ = [E_u_ ⊗ E_u_]^+^ = A_1g_ ⊕ B_1g_ ⊕ B_2g_ (8)

The full-symmetric a_1g_ coordinate does not change the symmetry of the structure (i.e., cannot be JT active) and the kernels

K(D_4h_, b_1g_) = D_2h_(C_2_′) (9)

K(D_4h_, b_2g_) = D_2h_(C_2_″) (10)

do not explain the existence of the optimized D_2_ structure. Therefore, the method of the epikernel principle [5,6] cannot explain the D_4h_ → D_2_ symmetry descent.

**Figure 5:**
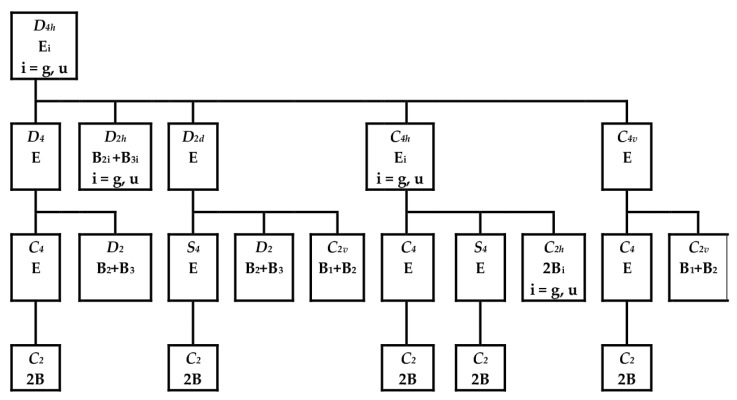
Possible symmetry descent paths of D_4h_ structures in double degenerate electron states [9]. The top and bottom lines of individual rectangles denote symmetry groups and the corresponding irreducible representations, respectively.

The method of step-by-step symmetry descent [8,9] for the structures of the D_4h_ symmetry group in double degenerate electron states is based on the scheme in Figure 5. Except D_2h_, all immediate subgroups of the D_4h_ group (i.e., D_4_, D_2d_, C_4v_, and C_4h_) are JT unstable because of preserved electron degeneracy. After the removal of the C_4_ axis from the D_4_ group, we obtain its immediate subgroup D_2_, which is JT stable because the degeneracy is removed here. Alternatively, the D_2_ group can be obtained via symmetry descent through the JT unstable D_2d_ group with preserved electron degeneracy. The structures of the C_4_ and S_4_ symmetry groups preserve electron degeneracy and therefore cannot be stable. We have not found any stable structure of D_2h_, C_2v_, or C_2h_ symmetry groups through geometry optimization. Therefore, the stable D_2_ structures meet the condition of maximal groups for category II complexes. 

During the symmetry descent from D_4h_ to D_2_, the double degenerate representation is split into the nondegenerate B_2_ and B_3_ ones:

E_g_ or E_u_ (D_4h_) → E (D_4_ or D_2d_) → B_2_ ⊕ B_3_ (D_2_) (11)

None of these representations corresponds to the calculated ground electron state of the stable D_2_ structures (Table 4). This discrepancy can be explained using the above-mentioned HPJT effect, and the ground state of the D_2_ structure corresponds to the lower PJT interacting excited state in its supergroup. In our case, the situation is complicated by the fact that D_2_ is not an immediate subgroup of the D_4h_ group. Therefore, there are three possible two-step symmetry descent paths:

(12) $$D_{4h}\;\overset{a_{1u}}{\to }\;D_{4}\;\overset{b_{1}\;{or\;b}_{2}}{\to }\;D_{2}$$

(13) $$D_{4h}\;\overset{b_{1g}{\;or\;b}_{2g}}{\to }\;D_{2h}\;\overset{a_{u}}{\to }\;D_{2}$$

(14) $$D_{4h}\;\overset{b_{1u}{\;or\;b}_{2u}}{\to }\;D_{2d}\;\overset{b_{1}}{\to }\;D_{2}$$

Based on group–subgroup relations, we can assign the electron state representations of the D_2_ symmetry group to the corresponding D_4_, D_2h_, or D_2d_ ones despite the several alternatives. The same holds for the D_4h_ to D_4_, D_2h_, or D_2d_ symmetry descent. For known representations of ground states and JT active coordinates, it might be possible to determine the HPJT excited-state representations according to Equation (4). For the two-step symmetry descent, there are too many possibilities where almost all excited-state representations (except two-dimensional) might be involved. Therefore, we shall not deal with this problem.

## 4. Methods

We have performed geometry optimization of the complexes ^m^[dmaphPcAg]^q^ with charges q = +1 → −2 in the two lowest spin states (singlet to quartet) with spin multiplicities m within the D_4h_ symmetry and its subgroups. In agreement with our previous study [1], B3LYP hybrid functional [12], GD3 dispersion correction [13], cc-pVDZ-PP pseudopotential and basis set for Ag [14], and cc-pVDZ basis sets for remaining atoms [15] were used. The optimized structures were tested on the number of imaginary vibrations. Excited states (up to 50) were calculated for every optimized structure using time-dependent DFT (TD-DFT) treatment [16,17], analogously to our previous studies [1,18,19]. All calculations were performed with Gaussian16 (Revision B.01) software [20]. MOLDRAW (Release 2.0, https://www.moldraw.software.informer.com, accessed on 9 September 2019) software [21] was used for visualization and geometry modification purposes. Finally, a group-theoretical analysis of the obtained results was carried out using the methods of the epikernel principle [5,6,7] and of step-by-step symmetry descent [8,9].

The B3LYP functional is most frequently used in quantum-chemical studies and produces relatively reliable excitation energies. The basis sets used are restricted by our technical capabilities.

## 5. Conclusions

To shed more light on the photopolymerization action of the Ag(II) complex with dimethylamino phenyl-substituted phthalocyanine ^2^[dmaphPcAg]^0^, we have performed a quantum-chemical model study of possible reaction intermediates [dmaphPcAg]^q^ with charges q = +1 → −2, with the aim to explain the possible role of the JT effect. Group-theoretical analysis of the obtained results shows that the complexes under study are of two categories.

The stable structures of maximal symmetry of ^1^[dmaphPcAg]^+^, ^3^[dmaphPcAg]^+^, ^2^[dmaphPcAg]^0^, and ^4^[dmaphPcAg]^2−^ complexes (category I) correspond to the D_4_ group as a consequence of the PJT effect within the unstable D_4h_ structure. On the other hand, complexes ^4^[dmaphPcAg]^0^, ^1^[dmaphPcAg]^−^, ^3^[dmaphPcAg]^−^, and ^2^[dmaphPcAg]^2−^ (category II) with double degenerate electron ground states in (JT unstable) D_4h_ symmetry structures undergo a symmetry descent to stable structures corresponding to maximal D_2_ symmetry, not because of a simple JT effect but due to HPJT effect. The most stable reaction intermediate in the supposed photoinitiation reaction [1] is surprisingly ^3^[dmaphPcAg]^−^ (a singlet electron state was expected). Therefore, the reduction of the ^2^[dmaphPcAg]^0^ photoinitiator (D_4_ symmetry) to the ^3^[dmaphPcAg]^−^ intermediate (D_2_ symmetry) must be significantly affected by vibronic interactions (PJT and HPJT effects), primarily the reaction barrier height and reaction equilibrium. Moreover, the reactivity of ^3^[dmaphPcAg]^−^ is supported by the non-equal spin density at nitrogen atoms of the D_2_ structure (doubled at one pair of these atoms; see Table S2 in Supplementary Materials). Nevertheless, their exact influence on reaction rates and thermodynamics must be investigated in solutions.

The presented study shows how group-theoretical treatment can be profitable through solving chemical problems for large molecules. The method of epikernel principle [5,6,7] is restricted to JT active coordinates based on perturbation theory and thus grants incomplete results, but it can also be used for the PJT effect. The method of step-by-step symmetry descent [8,9] based on splitting degenerate electron states during symmetry descent is more universal but not suitable for the PJT effect. We have shown the usefulness of the combination of both methods. Further theoretical studies in this field are welcome.

## Figures and Tables

**Table 1:** Charge q, spin multiplicity m, symmetry group G, representation of the ground electron state Γ_0_, DFT energy *E*_DFT_, JT stabilization energy *E*_JT_, representations Λ_im_ and wavenumbers ν_im_ of imaginary vibrations, kernel K(D_4h_, Λ_im_) and epikernel K(D_4h_, Λ_im_) subgroups of D_4h_, and representations of relevant PJT excited states Γ_exc_ of ^m^[dmaphPc]^q^ complexes under study (the preserved symmetry elements in the kernel and epikernel subgroups are in parentheses). The most stable structure is shown in bold.

q	m	G	Γ_0_	*E*_DFT_ [Hartree]	*E*_JT_ [eV]	Λ_im_	ν_im_ [cm^−1^]	K(D_4h_, Λ_im_)	E(D_4h_, Λ_im_)	Γ_exc_
+1	1	D_4h_	^1^A_1g_	−4734.55067	-	b_1u_	−48	D_2d_(C_2_′)		B_1u_
						2e_g_	−47, −31	C_1_	C_2h_(C_2_′), C_2h_(C_2_″)	E_g_
						a_1u_	−46	D_4_		A_1u_
						a_2u_	−31	C_4v_		A_2u_
						b_2u_	−31	D_2d_(C_2_″)		B_2u_
+1	1	D_4_	^1^A_1_	−4734.58352	0.894	-	-			
+1	3	D_4h_	^3^B_1u_	−4734.54698	-	b_1u_	−18	D_2d_(C_2_′)		A_1g_
						e_g_	−18	C_1_	C_2h_(C_2_′), C_2h_(C_2_″)	E_u_
						a_1u_	−18	D_4_		B_1g_
+1	3	D_4_	^3^B_1_	−4734.58550	1.048	-	-			
0	2	D_4h_	^2^B_1g_	−4734.74708	-	b_1u_	−42	D_2d_(C_2_′)		A_1u_
						e_g_	−42	C_1_	C_2h_(C_2_′), C_2h_(C_2_″)	E_g_
						a_1u_	−41	D_4_		B_1u_
						a_2u_	−23	C_4v_		B_2u_
						3b_2u_	−23(3×)	D_2d_(C_2_″)		A_2u_
0	2	D_4_	^2^B_1_	−4734.77283	0.701	-	-			
0	4	D_2_	^4^B_2_	−4734.73113	unknown	-	-			
−1	1	D_2_	^1^A	−4734.79846	unknown	-	-			
**−1**	**3**	**D_2_**	**^3^B_2_**	**−4734.83355**	unknown	-	-			
−2	2	D_2_	^2^B_1_	−4734.80520	unknown	-	-			
−2	4	D_4h_	^4^B_1u_	−4734.77355	-	b_1u_	−47	D_2d_(C_2_′)		A_1g_
						2e_g_	−46, −27	C_1_	C_2h_(C_2_′), C_2h_(C_2_″)	E_u_
						a_1u_	−45	D_4_		B_1g_
						a_2u_	−27	C_4v_		B_2g_
						b_2u_	−27	D_2d_(C_2_″)		A_2g_
−2	4	D_4_	^4^B_1_	−4734.80581	0.878	-	-			

**Table 2:** Charge q, spin multiplicity m, electron configuration, and ground electron state representation Γ_0_ of studied ^m^[dmaphPc]^q^ complexes of D_4h_ symmetry group.

q	m	Electron Configuration	Γ_0_
+1	1	…(b_2g_)^2^(e_u_)^4^(a_2g_)^2^(b_1g_)^0^(e_g_)^0^(b_1u_)^0^…	^1^A_1g_
+1	3	α: …(b_1g_)^1^(a_2g_)^1^(e_u_)^2^(b_2g_)^1^(e_g_)^0^(b_1u_)^0^… β: …(e_u_)^2^(b_2g_)^1^(a_1u_)^0^(e_g_)^0^(b_1g_)^0^(b_1u_)^0^…	^3^B_1u_
0	2	α:…(e_u_)^2^(b_2g_)^1^(b_1g_)^1^(a_1u_)^1^(e_g_)^0^(b_1u_)^0^… β:…(e_u_)^2^(b_2g_)^1^(a_1u_)^1^(e_g_)^0^(b_1g_)^0^(b_1u_)^0^…	^2^B_1g_
0	4	unknown	^4^E_g_ or ^4^E_u_
−1	1	unknown	^1^E_g_ or ^1^E_u_
−1	3	unknown	^3^E_g_ or ^3^E_u_
−2	2	unknown	^2^E_g_ or ^2^E_u_
−2	4	α:…(e_g_)^2^(b_2g_)^1^(a_1u_)^1^(b_1g_)^1^(e_g_)^2^(b_2u_)^0^… β:…(e_g_)^2^(a_2u_)^1^(a_1u_)^1^(e_g_)^0^(a_2u_)^0^(b_2u_)^0^…	^4^B_1u_

**Table 3:** Charge q, spin multiplicity m, symmetry group G, ground electron state representation Γ_0_, representations Γ_exc_, excitation energies *E*_exc_, and oscillator strengths *f* of the low excited electron states of the studied ^m^[dmaphPc]^q^ complexes in D_4h_ and D_4_ symmetry groups. The excited states that interact with the ground states are in bold.

q	m	G	Γ_0_	Γ_exc_	*E*_exc_ [eV]	*f*	G	Γ_0_	Γ_exc_	*E*_exc_ [eV]	*f*
+1	1	D_4h_	^1^A_1g_	1^1^A_2g_	0.156	0.000	D_4_	^1^A_1_	1^1^B_1_	0.167	0.000
				1^1^E_u_	0.158	0.002			1^1^B_2_	0.255	0.000
				1^1^B_2g_	0.159	0.000			1^1^E	0.259	0.000
				1^1^B_1g_	0.349	0.000			1^1^A_2_	0.267	0.000
				2^1^E_u_	0.353	0.081			2^1^E	0.631	0.014
				1^1^B_1u_	0.377	0.000			1^1^A_1_	0.642	0.000
				1^1^A_1g_	0.487	0.000			2^1^B_1_	0.858	0.000
				1^1^E_g_	1.010	0.000			3^1^E	1.187	0.050
				**1^1^A_1u_**	**1.010**	**0.000**			**2^1^A_1_**	**1.192**	**0.001**
				1^1^B_1u_	1.010	0.000			2^1^A_2_	1.200	0.000
+1	3	D_4h_	^3^B_1u_	1^3^B_2g_	0.011	0.000	D_4_	^3^B_1_	1^3^E	0.188	0.007
				1^3^A_2g_	0.015	0.001			1^3^B_2_	0.190	0.000
				1^3^E_u_	0.016	0.001			1^3^A_2_	0.195	0.000
				**1^3^A_1g_**	**0.235**	**0.000**			**1^3^A_1_**	**0.600**	**0.000**
				2^3^E_u_	0.235	0.014			2^3^E	0.617	0.298
				1^3^B_2u_	0.239	0.000			1^3^B_1_	0.779	0.000
				2^3^E_u_	1.255	0.000			3^3^E	1.244	0.001
				3^3^E_u_	1.327	0.003			4^3^E	1.391	0.033
				1^3^E_g_	1.493	0.008			2^3^B_1_	1.400	0.000
0	2	D_4h_	^2^B_1g_	1^2^E_u_	1.195	0.000	D_4_	^2^B_1_	1^2^E	1.157	0.000
				1^2^E_g_	1.306	0.000			2^2^E	1.305	0.000
				**1^2^B_1u_**	**2.014**	**0.000**			3^2^E	1.901	0.624
				2^2^E_u_	2.050	0.710			1^2^B_2_	1.942	0.000
				2^2^E_g_	2.140	0.000			1^2^A_2_	1.944	0.000
				1^2^B_2u_	2.140	0.000			4^2^E	1.956	0.004
				1^2^A_2u_	2.140	0.000			2^2^A_2_	1.969	0.000
				3^2^E_g_	2.163	0.000			1^2^A_1_	2.055	0.000
				2^2^B_2u_	2.164	0.000			3^2^A_2_	2.056	0.000
				2^2^A_2u_	2.164	0.000			**1^2^B_1_**	**2.059**	**0.001**
−2	4	D_4h_	^4^B_1u_	1^4^E_g_	0.957	0.000	D_4_	^4^B_1_	1^4^E	0.888	0.191
				1^4^A_2u_	0.976	0.000			1^4^B_1_	1.356	0.000
				1^4^B_2u_	0.978	0.000			2^4^E	1.360	0.010
				2^4^E_g_	1.025	0.000			1^4^A_1_	1.360	0.000
				1^4^A_1u_	1.042	0.000			3^4^E	1.396	0.010
				1^4^B_1u_	1.048	0.000			1^4^A_2_	1.415	0.000
				3^4^E_g_	1.118	0.110			1^4^B_2_	1.417	0.000
				1^4^A_1g_	1.495	0.000			4^4^E	1.436	0.012
				**1^4^B_1g_**	**1.495**	**0.000**			2^4^B_2_	1.472	0.001
				1^4^E_u_	1.496	0.000			2^4^A_2_	1.481	0.000
				2^4^A_1u_	1.501	0.000			**3^4^B_1_**	**1.523**	**0.000**

**Table 4:** Charge q, spin multiplicity m, representations of the ground electron state Γ_0_ and of the excited states Γ_exc_, excitation energies *E*_exc_, and oscillator strengths *f* of low excited electron states of stable ^m^[dmaphPc]^q^ complexes under study in the D_2_ symmetry group. The irreducible representations obtained through splitting the two-dimensional representations of the degenerate ground state in D_4h_ structures are in bold.

q	m	Γ_0_	Γ_exc_	*E*_exc_ [eV]	*f*	q	m	Γ_0_	Γ_exc_	*E*_exc_ [eV]	*f*
0	4	^4^B_2_	1^4^B_1_	0.349	0.000	−1	1	^1^A	**1^1^B_2_**	**0.523**	**0.000**
			**1^4^B_3_**	**0.814**	**0.012**				1^1^B_1_	0.539	0.000
			2^4^B_1_	0.829	0.000				**1^1^B_3_**	**0.815**	**0.000**
			**1^4^B_2_**	**0.927**	**0.006**				2^1^B_2_	1.337	0.023
			3^4^B_1_	0.948	0.000				2^1^B_3_	1.478	0.028
			2^4^B_3_	1.253	0.000				3^1^B_2_	1.551	0.005
			2^4^B_2_	1.254	0.213				4^1^B_2_	1.793	0.809
			1^4^A	1.291	0.000				3^1^B_3_	1.965	0.018
			3^4^B_3_	1.356	0.164				1^1^A	2.056	0.000
			2^4^A	1.423	0.000				4^1^B_3_	2.097	0.011
−1	3	^3^B_2_	1^3^B_1_	0.272	0.000	−2	2	^2^B_1_	1^2^B_1_	0.048	0.000
			1^3^B_3_	1.256	0.000				2^2^B_1_	0.377	0.000
			**1^3^B_2_**	**1.350**	**0.021**				1^2^B_2_	0.900	0.007
			**2^3^B_3_**	**1.356**	**0.014**				**2^2^B_2_**	**1.095**	**0.283**
			2^3^B_2_	1.796	0.792				**1^2^B_3_**	**1.112**	**0.306**
			3^3^B_3_	1.835	0.007				3^2^B_2_	1.168	0.000
			4^3^B_3_	1.987	0.012				1^2^A	1.343	0.000
			1^3^A	1.993	0.000				4^2^B_2_	1.348	0.002
			2^3^B_1_	2.000	0.000				2^2^A	1.362	0.000
			2^3^A	2.092	0.000				2^2^B_3_	1.367	0.002

## Data Availability

Data are contained within the article.

## Supplementary Materials

The following supporting information can be downloaded at: https://www.mdpi.com/article/10.3390/molecules28207019/s1, Table S1. Relations between the irreducible representations of the parent group D_4h_ and of its D_4_ and D_2_ subgroups. Table S2. Charge q, spin multiplicity m, symmetry group G, Mulliken atomic charges and spin populations of Ag and pyrrole N atoms in ^m^[dmaphPc]^q^ complexes under study. Tables S3–S14. Atom coordinates of ^m^[dmaphPc]^q^ in D_4h_, D_4_ or D_2_ symmetry (in Å).
