# Supplementary material for: On the Jahn–Teller Effect in Silver Complexes of Dimethyl Amino Phenyl Substituted Phthalocyanine
Source: Molecules. 2023 Oct 10;28(20):7019. doi: 10.3390/molecules28207019 (PMC10609293; doi:10.3390/molecules28207019)
Supplement: Supplementary file 1 [file molecules-28-07019-s001.zip › molecules-2630241-supplementary.pdf]

On the Jahn-Teller Effect in Silver Complexes of Dimethyl Amino Phenyl Substituted Phthalocyanine

Martin Breza

Department of Physical Chemistry, Slovak Technical University, Radlinskeho 9, SK-81237 Bratislava, Slovakia

Supplementary information

Table S1. Relations between the irreducible representations of the parent group D_{4h} and of its D_4 and D_2 subgroups (the preserved two-fold rotation axes are in parentheses).

D_{4h}	D_4	$D_2(C_2')$	$D_2(C_2'')$
A_{1g}	A_1	A	A
A_{2g}	A_2	B_1	B_1
B_{1g}	B_1	A	B_1
B_{2g}	B_2	B_1	A
E_g	E	$B_2 + B_3$	$B_2 + B_3$
A_{1u}	A_1	A	A
A_{2u}	A_2	B_1	B_1
B_{1u}	B_1	A	B_1
B_{2u}	B_2	B_1	A
E_u	E	$B_2 + B_3$	$B_2 + B_3$

Table S2. Charge q, spin multiplicity m, symmetry group G, Mulliken atomic charges and spin populations of Ag and pyrrole N atoms in $^{m}[dmaphPc]^{q}$ complexes under study.

			Atomic charge		Spin population	
q	m	G	Ag	N	Ag	N
+1	1	D_{4h}	0.771	-0.430(4×)	-	-
		D_4	0.784	-0.439(4×)	-	-
+1	3	D_{4h}	0.616	-0.392(4×)	0.426	0.095(4×)
		D_4	0.605	-0.392(4×)	0.426	0.107(4×)
0	2	D_{4h}	0.584	-0.213 (4×)	0.422	0.139(4×)
		D_4	0.583	-0.383(4×)	0.422	0.140(4×)
0	4	D_2	0.578	-0.376(2×)	0.426	0.060(2×)
				-0.416(2×)		0.196(2×)
-1	1	D_2	0.723	-0.410(2×)	-	-
				-0.478(2×)		
-1	3	D_2	0.549	-0.369(2×)	0.421	0.109(2×)
				-0.403(2×)		0.231(2×)
-2	2	D_2	0.520	-0.389(2×)	0.415	0.253(2×)
				-0.391(2×)		0.027(2×)
-2	4	D_{4h}	0.509	-0.388(4×)	0.416	0.205(4×)
		D_4	0.518	-0.389(4×)	0.417	0.201(4×)

Table S3. Atom coordinates of [1][dmaphPc]$^+$ in D_{4h} symmetry (in Å).

6	-1.136489	2.792603	0.000000
7	-2.401796	2.401796	0.000000
7	0.000000	2.004075	0.000000
6	1.136489	2.792603	0.000000
7	2.401796	2.401796	0.000000
6	2.792603	1.136489	0.000000
7	2.004075	-0.000000	0.000000
6	2.792603	-1.136489	-0.000000
6	-0.705331	4.180772	0.000000
6	0.705331	4.180772	0.000000
6	4.180772	0.705331	0.000000
6	4.180772	-0.705331	-0.000000
7	2.401796	-2.401796	0.000000
6	1.136489	-2.792603	0.000000
7	-0.000000	-2.004075	0.000000
6	-1.136489	-2.792603	-0.000000
6	0.705331	-4.180772	0.000000
6	-0.705331	-4.180772	-0.000000
7	-2.401796	-2.401796	-0.000000
6	-2.792603	-1.136489	0.000000
7	-2.004075	0.000000	0.000000
6	-2.792603	1.136489	-0.000000
6	-4.180772	-0.705331	0.000000
6	-4.180772	0.705331	-0.000000
6	-1.423449	5.377536	0.000000
6	-0.714694	6.587196	0.000000
6	0.714694	6.587196	0.000000
6	1.423449	5.377536	0.000000
6	-1.466346	7.876645	0.000000
6	-1.828973	8.504849	1.198974
6	-2.520461	9.713448	1.209321
6	-2.876212	10.361775	0.000000
6	-2.520461	9.713448	-1.209321
6	-1.828973	8.504849	-1.198974
6	1.466346	7.876645	0.000000
6	1.828973	8.504849	-1.198974
6	2.520461	9.713448	-1.209321
6	2.876212	10.361775	0.000000
6	2.520461	9.713448	1.209321
6	1.828973	8.504849	1.198974
6	5.377536	1.423449	0.000000
6	6.587196	0.714694	0.000000
6	6.587196	-0.714694	-0.000000
6	5.377536	-1.423449	-0.000000
6	7.876645	1.466346	0.000000
6	8.504849	1.828973	1.198974
6	9.713448	2.520461	1.209321
6	10.361775	2.876212	0.000000
6	9.713448	2.520461	-1.209321

6	8.504849	1.828973	-1.198974
6	7.876645	-1.466346	-0.000000
6	8.504849	-1.828973	1.198974
6	9.713448	-2.520461	1.209321
6	10.361775	-2.876212	-0.000000
6	9.713448	-2.520461	-1.209321
6	8.504849	-1.828973	-1.198974
6	1.423449	-5.377536	0.000000
6	0.714694	-6.587196	0.000000
6	-0.714694	-6.587196	-0.000000
6	-1.423449	-5.377536	-0.000000
6	1.466346	-7.876645	0.000000
6	1.828973	-8.504849	1.198974
6	2.520461	-9.713448	1.209321
6	2.876212	-10.361775	0.000000
6	2.520461	-9.713448	-1.209321
6	1.828973	-8.504849	-1.198974
6	-1.466346	-7.876645	-0.000000
6	-1.828973	-8.504849	-1.198974
6	-2.520461	-9.713448	-1.209321
6	-2.876212	-10.361775	-0.000000
6	-2.520461	-9.713448	1.209321
6	-1.828973	-8.504849	1.198974
6	-5.377536	-1.423449	0.000000
6	-6.587196	-0.714694	0.000000
6	-6.587196	0.714694	-0.000000
6	-5.377536	1.423449	-0.000000
6	-7.876645	-1.466346	0.000000
6	-8.504849	-1.828973	-1.198974
6	-9.713448	-2.520461	-1.209321
6	-10.361775	-2.876212	0.000000
6	-9.713448	-2.520461	1.209321
6	-8.504849	-1.828973	1.198974
6	-7.876645	1.466346	-0.000000
6	-8.504849	1.828973	-1.198974
6	-9.713448	2.520461	-1.209321
6	-10.361775	2.876212	-0.000000
6	-9.713448	2.520461	1.209321
6	-8.504849	1.828973	1.198974
1	-2.513867	5.373744	0.000000
1	2.513867	5.373744	0.000000
1	-1.562274	8.039655	2.150904
1	-2.778658	10.153945	2.170787
7	-3.538918	11.576892	0.000000
1	-2.778658	10.153945	-2.170787
1	-1.562274	8.039655	-2.150904
1	1.562274	8.039655	-2.150904
1	2.778658	10.153945	-2.170787
7	3.538918	11.576892	0.000000
1	2.778658	10.153945	2.170787
1	1.562274	8.039655	2.150904

1	5.373744	2.513867	0.000000
1	5.373744	-2.513867	-0.000000
1	8.039655	1.562274	2.150904
1	10.153945	2.778658	2.170787
7	11.576892	3.538918	0.000000
1	10.153945	2.778658	-2.170787
1	8.039655	1.562274	-2.150904
1	8.039655	-1.562274	2.150904
1	10.153945	-2.778658	2.170787
7	11.576892	-3.538918	-0.000000
1	10.153945	-2.778658	-2.170787
1	8.039655	-1.562274	-2.150904
1	2.513867	-5.373744	0.000000
1	-2.513867	-5.373744	-0.000000
1	1.562274	-8.039655	2.150904
1	2.778658	-10.153945	2.170787
7	3.538918	-11.576892	0.000000
1	2.778658	-10.153945	-2.170787
1	1.562274	-8.039655	-2.150904
1	-1.562274	-8.039655	-2.150904
1	-2.778658	-10.153945	-2.170787
7	-3.538918	-11.576892	-0.000000
1	-2.778658	-10.153945	2.170787
1	-1.562274	-8.039655	2.150904
1	-5.373744	-2.513867	0.000000
1	-5.373744	2.513867	-0.000000
1	-8.039655	-1.562274	-2.150904
1	-10.153945	-2.778658	-2.170787
7	-11.576892	-3.538918	0.000000
1	-10.153945	-2.778658	2.170787
1	-8.039655	-1.562274	2.150904
1	-8.039655	1.562274	-2.150904
1	-10.153945	2.778658	-2.170787
7	-11.576892	3.538918	-0.000000
1	-10.153945	2.778658	2.170787
1	-8.039655	1.562274	2.150904
6	4.023509	-12.131414	-1.250598
6	4.023509	-12.131414	1.250598
6	-4.023509	-12.131414	-1.250598
6	-4.023509	-12.131414	1.250598
6	-12.131414	-4.023509	-1.250598
6	-12.131414	-4.023509	1.250598
6	-12.131414	4.023509	1.250598
6	-12.131414	4.023509	-1.250598
6	-4.023509	12.131414	1.250598
6	-4.023509	12.131414	-1.250598
6	4.023509	12.131414	1.250598
6	4.023509	12.131414	-1.250598
6	12.131414	4.023509	1.250598
6	12.131414	4.023509	-1.250598
6	12.131414	-4.023509	1.250598

6	12.131414	-4.023509	-1.250598
1	3.195361	-12.315782	-1.956647
1	4.755940	-11.470636	-1.755074
1	4.511021	-13.094964	-1.055383
1	4.511021	-13.094964	1.055383
1	4.755940	-11.470636	1.755074
1	3.195361	-12.315782	1.956647
1	-4.755940	-11.470636	-1.755074
1	-3.195361	-12.315782	-1.956647
1	-4.511021	-13.094964	-1.055383
1	-4.511021	-13.094964	1.055383
1	-3.195361	-12.315782	1.956647
1	-4.755940	-11.470636	1.755074
1	-12.315782	-3.195361	-1.956647
1	-11.470636	-4.755940	-1.755074
1	-13.094964	-4.511021	-1.055383
1	-11.470636	-4.755940	1.755074
1	-12.315782	-3.195361	1.956647
1	-13.094964	-4.511021	1.055383
1	-12.315782	3.195361	1.956647
1	-11.470636	4.755940	1.755074
1	-13.094964	4.511021	1.055383
1	-11.470636	4.755940	-1.755074
1	-12.315782	3.195361	-1.956647
1	-13.094964	4.511021	-1.055383
1	-4.755940	11.470636	1.755074
1	-3.195361	12.315782	1.956647
1	-4.511021	13.094964	1.055383
1	-3.195361	12.315782	-1.956647
1	-4.755940	11.470636	-1.755074
1	-4.511021	13.094964	-1.055383
1	3.195361	12.315782	1.956647
1	4.755940	11.470636	1.755074
1	4.511021	13.094964	1.055383
1	4.755940	11.470636	-1.755074
1	3.195361	12.315782	-1.956647
1	4.511021	13.094964	-1.055383
1	11.470636	4.755940	1.755074
1	12.315782	3.195361	1.956647
1	13.094964	4.511021	1.055383
1	12.315782	3.195361	-1.956647
1	11.470636	4.755940	-1.755074
1	13.094964	4.511021	-1.055383
1	12.315782	-3.195361	1.956647
1	11.470636	-4.755940	1.755074
1	13.094964	-4.511021	1.055383
1	11.470636	-4.755940	-1.755074
1	12.315782	-3.195361	-1.956647
1	13.094964	-4.511021	-1.055383
47	0.000000	0.000000	0.000000

Table S4. Atom coordinates of [1][dmaphPc]$^+$ in D_4 symmetry (in Å).

C	-1.137505072097	2.790913541087	-0.000675991081
N	-2.403191801854	2.403191801854	0.000000000000
N	-0.000000000000	1.998518150637	0.000000000000
C	1.137505072097	2.790913541087	0.000675991081
N	2.403191801854	2.403191801854	0.000000000000
C	2.790913541087	1.137505072097	-0.000675991081
N	1.998518150637	0.000000000000	0.000000000000
C	2.790913541087	-1.137505072097	0.000675991081
C	-0.705064333981	4.174818155681	-0.002456594857
C	0.705064333981	4.174818155681	0.002456594857
C	4.174818155681	0.705064333981	-0.002456594857
C	4.174818155681	-0.705064333981	0.002456594857
N	2.403191801854	-2.403191801854	0.000000000000
C	1.137505072097	-2.790913541087	-0.000675991081
N	0.000000000000	-1.998518150637	0.000000000000
C	-1.137505072097	-2.790913541087	0.000675991081
C	0.705064333981	-4.174818155681	-0.002456594857
C	-0.705064333981	-4.174818155681	0.002456594857
N	-2.403191801854	-2.403191801854	0.000000000000
C	-2.790913541087	-1.137505072097	-0.000675991081
N	-1.998518150637	-0.000000000000	0.000000000000
C	-2.790913541087	1.137505072097	0.000675991081
C	-4.174818155681	-0.705064333981	-0.002456594857
C	-4.174818155681	0.705064333981	0.002456594857
C	-1.417167510509	5.372638552914	0.010193366783
C	-0.720453663762	6.592889532341	0.017655729516
C	0.720453663762	6.592889532341	-0.017655729516
C	1.417167510508	5.372638552914	-0.010193366783
C	-1.515999794293	7.842862251078	0.099818538921
C	-2.639469961998	8.040063876318	-0.721651152363
C	-3.416580092692	9.191901928681	-0.644755168924
C	-3.108168442102	10.218612801634	0.283190596756
C	-1.987761315223	10.007867582375	1.129274512196
C	-1.215945491202	8.858348026355	1.026732241242
C	1.515999794293	7.842862251078	-0.099818538921
C	2.639469961998	8.040063876319	0.721651152363
C	3.416580092692	9.191901928681	0.644755168924
C	3.108168442101	10.218612801634	-0.283190596756
C	1.987761315222	10.007867582375	-1.129274512196
C	1.215945491202	8.858348026355	-1.026732241242
C	5.372638552914	1.417167510509	0.010193366783
C	6.592889532341	0.720453663762	0.017655729516
C	6.592889532341	-0.720453663762	-0.017655729516
C	5.372638552914	-1.417167510508	-0.010193366783
C	7.842862251078	1.515999794293	0.099818538921
C	8.040063876318	2.639469961998	-0.721651152363
C	9.191901928681	3.416580092692	-0.644755168924
C	10.218612801634	3.108168442102	0.283190596756
C	10.007867582375	1.987761315223	1.129274512196

C	8.858348026355	1.215945491202	1.026732241242
C	7.842862251078	-1.515999794293	-0.099818538921
C	8.858348026355	-1.215945491202	-1.026732241242
C	10.007867582375	-1.987761315222	-1.129274512196
C	10.218612801634	-3.108168442101	-0.283190596756
C	9.191901928681	-3.416580092692	0.644755168924
C	8.040063876319	-2.639469961998	0.721651152363
C	1.417167510509	-5.372638552914	0.010193366783
C	0.720453663762	-6.592889532341	0.017655729516
C	-0.720453663762	-6.592889532341	-0.017655729516
C	-1.417167510508	-5.372638552914	-0.010193366783
C	1.515999794293	-7.842862251078	0.099818538921
C	2.639469961998	-8.040063876318	-0.721651152363
C	3.416580092692	-9.191901928681	-0.644755168924
C	3.108168442102	-10.218612801634	0.283190596756
C	1.987761315223	-10.007867582375	1.129274512196
C	1.215945491202	-8.858348026355	1.026732241242
C	-1.515999794293	-7.842862251078	-0.099818538921
C	-2.639469961998	-8.040063876319	0.721651152363
C	-3.416580092692	-9.191901928681	0.644755168924
C	-3.108168442101	-10.218612801634	-0.283190596756
C	-1.987761315222	-10.007867582375	-1.129274512196
C	-1.215945491202	-8.858348026355	-1.026732241242
C	-5.372638552914	-1.417167510509	0.010193366783
C	-6.592889532341	-0.720453663762	0.017655729516
C	-6.592889532341	0.720453663762	-0.017655729516
C	-5.372638552914	1.417167510508	-0.010193366783
C	-7.842862251078	-1.515999794293	0.099818538921
C	-8.858348026355	-1.215945491202	1.026732241242
C	-10.007867582375	-1.987761315223	1.129274512196
C	-10.218612801634	-3.108168442102	0.283190596756
C	-9.191901928681	-3.416580092692	-0.644755168924
C	-8.040063876318	-2.639469961998	-0.721651152363
C	-7.842862251078	1.515999794293	-0.099818538921
C	-8.040063876319	2.639469961998	0.721651152363
C	-9.191901928681	3.416580092692	0.644755168924
C	-10.218612801634	3.108168442101	-0.283190596756
C	-10.007867582375	1.987761315222	-1.129274512196
C	-8.858348026355	1.215945491202	-1.026732241242
H	-2.506401172486	5.359231949407	0.056713397689
H	2.506401172486	5.359231949407	-0.056713397689
H	-2.901694497197	7.280809252439	-1.462651532594
H	-4.264708327057	9.291001481540	-1.320061359449
N	-3.861040301329	11.373147142855	0.359172448995
H	-1.712549584477	10.748920279859	1.877695564731
H	-0.359641288361	8.741268136708	1.692390721139
H	2.901694497197	7.280809252439	1.462651532594
H	4.264708327057	9.291001481540	1.320061359449
N	3.861040301329	11.373147142855	-0.359172448995
H	1.712549584477	10.748920279859	-1.877695564731
H	0.359641288361	8.741268136708	-1.692390721139

H	5.359231949407	2.506401172486	0.056713397689
H	5.359231949407	-2.506401172486	-0.056713397689
H	7.280809252439	2.901694497197	-1.462651532594
H	9.291001481540	4.264708327057	-1.320061359449
N	11.373147142855	3.861040301329	0.359172448995
H	10.748920279859	1.712549584477	1.877695564731
H	8.741268136708	0.359641288361	1.692390721139
H	8.741268136708	-0.359641288361	-1.692390721139
H	10.748920279859	-1.712549584477	-1.877695564731
N	11.373147142855	-3.861040301329	-0.359172448995
H	9.291001481540	-4.264708327056	1.320061359449
H	7.280809252439	-2.901694497197	1.462651532594
H	2.506401172486	-5.359231949407	0.056713397689
H	-2.506401172486	-5.359231949407	-0.056713397689
H	2.901694497197	-7.280809252439	-1.462651532594
H	4.264708327057	-9.291001481540	-1.320061359449
N	3.861040301329	-11.373147142855	0.359172448995
H	1.712549584477	-10.748920279859	1.877695564731
H	0.359641288361	-8.741268136708	1.692390721139
H	-2.901694497197	-7.280809252439	1.462651532594
H	-4.264708327056	-9.291001481540	1.320061359449
N	-3.861040301329	-11.373147142855	-0.359172448995
H	-1.712549584477	-10.748920279859	-1.877695564731
H	-0.359641288361	-8.741268136708	-1.692390721139
H	-5.359231949407	-2.506401172486	0.056713397689
H	-5.359231949407	2.506401172486	-0.056713397689
H	-8.741268136708	-0.359641288361	1.692390721139
H	-10.748920279859	-1.712549584477	1.877695564731
N	-11.373147142855	-3.861040301329	0.359172448995
H	-9.291001481540	-4.264708327057	-1.320061359449
H	-7.280809252439	-2.901694497197	-1.462651532594
H	-7.280809252439	2.901694497197	1.462651532594
H	-9.291001481540	4.264708327056	1.320061359449
N	-11.373147142855	3.861040301329	-0.359172448995
H	-10.748920279859	1.712549584477	-1.877695564731
H	-8.741268136708	0.359641288361	-1.692390721139
C	3.575901459880	-12.358838105477	1.386295096953
C	5.063592533605	-11.507133938638	-0.442584316554
C	-5.063592533605	-11.507133938638	0.442584316554
C	-3.575901459880	-12.358838105477	-1.386295096953
C	-12.358838105477	-3.575901459880	1.386295096953
C	-11.507133938638	-5.063592533605	-0.442584316554
C	-12.358838105477	3.575901459880	-1.386295096953
C	-11.507133938638	5.063592533605	0.442584316554
C	-5.063592533605	11.507133938638	-0.442584316554
C	-3.575901459880	12.358838105477	1.386295096953
C	3.575901459880	12.358838105477	-1.386295096953
C	5.063592533605	11.507133938638	0.442584316554
C	11.507133938638	5.063592533605	-0.442584316554
C	12.358838105477	3.575901459880	1.386295096953
C	12.358838105477	-3.575901459880	-1.386295096953

C	11.507133938638	-5.063592533605	0.442584316554
H	2.546685325071	-12.748794236795	1.298261162667
H	3.695691315816	-11.950723434938	2.408340316080
H	4.263123467132	-13.206962003106	1.276007889325
H	5.521034131866	-12.485701820654	-0.250624572608
H	5.813503801218	-10.726222871511	-0.211264744218
H	4.839682474753	-11.448760070171	-1.522466400970
H	-5.813503801218	-10.726222871511	0.211264744218
H	-4.839682474753	-11.448760070171	1.522466400970
H	-5.521034131866	-12.485701820654	0.250624572608
H	-4.263123467132	-13.206962003106	-1.276007889325
H	-2.546685325071	-12.748794236795	-1.298261162667
H	-3.695691315815	-11.950723434938	-2.408340316080
H	-12.748794236795	-2.546685325071	1.298261162667
H	-11.950723434938	-3.695691315816	2.408340316080
H	-13.206962003106	-4.263123467132	1.276007889325
H	-10.726222871511	-5.813503801218	-0.211264744218
H	-11.448760070171	-4.839682474753	-1.522466400970
H	-12.485701820654	-5.521034131866	-0.250624572608
H	-12.748794236795	2.546685325071	-1.298261162667
H	-11.950723434938	3.695691315815	-2.408340316080
H	-13.206962003106	4.263123467132	-1.276007889325
H	-10.726222871511	5.813503801218	0.211264744218
H	-11.448760070171	4.839682474753	1.522466400970
H	-12.485701820654	5.521034131866	0.250624572608
H	-5.813503801218	10.726222871511	-0.211264744218
H	-4.839682474753	11.448760070171	-1.522466400970
H	-5.521034131866	12.485701820654	-0.250624572608
H	-2.546685325071	12.748794236795	1.298261162667
H	-3.695691315816	11.950723434938	2.408340316080
H	-4.263123467132	13.206962003106	1.276007889325
H	2.546685325071	12.748794236795	-1.298261162667
H	3.695691315815	11.950723434938	-2.408340316080
H	4.263123467132	13.206962003106	-1.276007889325
H	5.813503801218	10.726222871511	0.211264744218
H	4.839682474753	11.448760070171	1.522466400970
H	5.521034131866	12.485701820654	0.250624572608
H	10.726222871511	5.813503801218	-0.211264744218
H	11.448760070171	4.839682474753	-1.522466400970
H	12.485701820654	5.521034131866	-0.250624572608
H	12.748794236795	2.546685325071	1.298261162667
H	11.950723434938	3.695691315816	2.408340316080
H	13.206962003106	4.263123467132	1.276007889325
H	12.748794236795	-2.546685325071	-1.298261162667
H	11.950723434938	-3.695691315815	-2.408340316080
H	13.206962003106	-4.263123467132	-1.276007889325
H	10.726222871511	-5.813503801218	0.211264744218
H	11.448760070171	-4.839682474753	1.522466400970
H	12.485701820654	-5.521034131866	0.250624572608
Ag	0.000000000000	-0.000000000000	0.000000000000

Table S5. Atom coordinates of [3][dmaphPc]+ in D_{4h} symmetry (in Å).

6	-1.134414	2.819815	0.000000
7	-2.406303	2.406303	0.000000
7	0.000000	2.051315	0.000000
6	1.134414	2.819815	0.000000
7	2.406303	2.406303	0.000000
6	2.819815	1.134414	0.000000
7	2.051315	-0.000000	0.000000
6	2.819815	-1.134414	-0.000000
6	-0.707298	4.225335	0.000000
6	0.707298	4.225335	0.000000
6	4.225335	0.707298	0.000000
6	4.225335	-0.707298	-0.000000
7	2.406303	-2.406303	0.000000
6	1.134414	-2.819815	0.000000
7	-0.000000	-2.051315	0.000000
6	-1.134414	-2.819815	-0.000000
6	0.707298	-4.225335	0.000000
6	-0.707298	-4.225335	-0.000000
7	-2.406303	-2.406303	-0.000000
6	-2.819815	-1.134414	0.000000
7	-2.051315	0.000000	0.000000
6	-2.819815	1.134414	-0.000000
6	-4.225335	-0.707298	0.000000
6	-4.225335	0.707298	-0.000000
6	-1.419798	5.417703	0.000000
6	-0.709943	6.637230	0.000000
6	0.709943	6.637230	0.000000
6	1.419798	5.417703	0.000000
6	-1.467697	7.922966	0.000000
6	-1.834451	8.548363	1.199026
6	-2.534798	9.751989	1.209117
6	-2.895531	10.397743	0.000000
6	-2.534798	9.751989	-1.209117
6	-1.834451	8.548363	-1.199026
6	1.467697	7.922966	0.000000
6	1.834451	8.548363	-1.199026
6	2.534798	9.751989	-1.209117
6	2.895531	10.397743	0.000000
6	2.534798	9.751989	1.209117
6	1.834451	8.548363	1.199026
6	5.417703	1.419798	0.000000
6	6.637230	0.709943	0.000000
6	6.637230	-0.709943	-0.000000
6	5.417703	-1.419798	-0.000000
6	7.922966	1.467697	0.000000
6	8.548363	1.834451	1.199026
6	9.751989	2.534798	1.209117
6	10.397743	2.895531	0.000000
6	9.751989	2.534798	-1.209117

6	8.548363	1.834451	-1.199026
6	7.922966	-1.467697	-0.000000
6	8.548363	-1.834451	1.199026
6	9.751989	-2.534798	1.209117
6	10.397743	-2.895531	-0.000000
6	9.751989	-2.534798	-1.209117
6	8.548363	-1.834451	-1.199026
6	1.419798	-5.417703	0.000000
6	0.709943	-6.637230	0.000000
6	-0.709943	-6.637230	-0.000000
6	-1.419798	-5.417703	-0.000000
6	1.467697	-7.922966	0.000000
6	1.834451	-8.548363	1.199026
6	2.534798	-9.751989	1.209117
6	2.895531	-10.397743	0.000000
6	2.534798	-9.751989	-1.209117
6	1.834451	-8.548363	-1.199026
6	-1.467697	-7.922966	-0.000000
6	-1.834451	-8.548363	-1.199026
6	-2.534798	-9.751989	-1.209117
6	-2.895531	-10.397743	-0.000000
6	-2.534798	-9.751989	1.209117
6	-1.834451	-8.548363	1.199026
6	-5.417703	-1.419798	0.000000
6	-6.637230	-0.709943	0.000000
6	-6.637230	0.709943	-0.000000
6	-5.417703	1.419798	-0.000000
6	-7.922966	-1.467697	0.000000
6	-8.548363	-1.834451	-1.199026
6	-9.751989	-2.534798	-1.209117
6	-10.397743	-2.895531	0.000000
6	-9.751989	-2.534798	1.209117
6	-8.548363	-1.834451	1.199026
6	-7.922966	1.467697	-0.000000
6	-8.548363	1.834451	-1.199026
6	-9.751989	2.534798	-1.209117
6	-10.397743	2.895531	-0.000000
6	-9.751989	2.534798	1.209117
6	-8.548363	1.834451	1.199026
1	-2.510577	5.416292	0.000000
1	2.510577	5.416292	0.000000
1	-1.564213	8.085624	2.151185
1	-2.795670	10.190699	2.170652
7	-3.567491	11.607795	0.000000
1	-2.795670	10.190699	-2.170652
1	-1.564213	8.085624	-2.151185
1	1.564213	8.085624	-2.151185
1	2.795670	10.190699	-2.170652
7	3.567491	11.607795	0.000000
1	2.795670	10.190699	2.170652
1	1.564213	8.085624	2.151185

1	5.416292	2.510577	0.000000
1	5.416292	-2.510577	-0.000000
1	8.085624	1.564213	2.151185
1	10.190699	2.795670	2.170652
7	11.607795	3.567491	0.000000
1	10.190699	2.795670	-2.170652
1	8.085624	1.564213	-2.151185
1	8.085624	-1.564213	2.151185
1	10.190699	-2.795670	2.170652
7	11.607795	-3.567491	-0.000000
1	10.190699	-2.795670	-2.170652
1	8.085624	-1.564213	-2.151185
1	2.510577	-5.416292	0.000000
1	-2.510577	-5.416292	-0.000000
1	1.564213	-8.085624	2.151185
1	2.795670	-10.190699	2.170652
7	3.567491	-11.607795	0.000000
1	2.795670	-10.190699	-2.170652
1	1.564213	-8.085624	-2.151185
1	-1.564213	-8.085624	-2.151185
1	-2.795670	-10.190699	-2.170652
7	-3.567491	-11.607795	-0.000000
1	-2.795670	-10.190699	2.170652
1	-1.564213	-8.085624	2.151185
1	-5.416292	-2.510577	0.000000
1	-5.416292	2.510577	-0.000000
1	-8.085624	-1.564213	-2.151185
1	-10.190699	-2.795670	-2.170652
7	-11.607795	-3.567491	0.000000
1	-10.190699	-2.795670	2.170652
1	-8.085624	-1.564213	2.151185
1	-8.085624	1.564213	-2.151185
1	-10.190699	2.795670	-2.170652
7	-11.607795	3.567491	-0.000000
1	-10.190699	2.795670	2.170652
1	-8.085624	1.564213	2.151185
6	4.057637	-12.157411	-1.250532
6	4.057637	-12.157411	1.250532
6	-4.057637	-12.157411	-1.250532
6	-4.057637	-12.157411	1.250532
6	-12.157411	-4.057637	-1.250532
6	-12.157411	-4.057637	1.250532
6	-12.157411	4.057637	1.250532
6	-12.157411	4.057637	-1.250532
6	-4.057637	12.157411	1.250532
6	-4.057637	12.157411	-1.250532
6	4.057637	12.157411	1.250532
6	4.057637	12.157411	-1.250532
6	12.157411	4.057637	1.250532
6	12.157411	4.057637	-1.250532
6	12.157411	-4.057637	1.250532

6	12.157411	-4.057637	-1.250532
1	3.231682	-12.348896	-1.957284
1	4.784779	-11.490230	-1.754401
1	4.553358	-13.116828	-1.055449
1	4.553358	-13.116828	1.055449
1	4.784779	-11.490230	1.754401
1	3.231682	-12.348896	1.957284
1	-4.784779	-11.490230	-1.754401
1	-3.231682	-12.348896	-1.957284
1	-4.553358	-13.116828	-1.055449
1	-4.553358	-13.116828	1.055449
1	-3.231682	-12.348896	1.957284
1	-4.784779	-11.490230	1.754401
1	-12.348896	-3.231682	-1.957284
1	-11.490230	-4.784779	-1.754401
1	-13.116828	-4.553358	-1.055449
1	-11.490230	-4.784779	1.754401
1	-12.348896	-3.231682	1.957284
1	-13.116828	-4.553358	1.055449
1	-12.348896	3.231682	1.957284
1	-11.490230	4.784779	1.754401
1	-13.116828	4.553358	1.055449
1	-11.490230	4.784779	-1.754401
1	-12.348896	3.231682	-1.957284
1	-13.116828	4.553358	-1.055449
1	-4.784779	11.490230	1.754401
1	-3.231682	12.348896	1.957284
1	-4.553358	13.116828	1.055449
1	-3.231682	12.348896	-1.957284
1	-4.784779	11.490230	-1.754401
1	-4.553358	13.116828	-1.055449
1	3.231682	12.348896	1.957284
1	4.784779	11.490230	1.754401
1	4.553358	13.116828	1.055449
1	4.784779	11.490230	-1.754401
1	3.231682	12.348896	-1.957284
1	4.553358	13.116828	-1.055449
1	11.490230	4.784779	1.754401
1	12.348896	3.231682	1.957284
1	13.116828	4.553358	1.055449
1	12.348896	3.231682	-1.957284
1	11.490230	4.784779	-1.754401
1	13.116828	4.553358	-1.055449
1	12.348896	-3.231682	1.957284
1	11.490230	-4.784779	1.754401
1	13.116828	-4.553358	1.055449
1	11.490230	-4.784779	-1.754401
1	12.348896	-3.231682	-1.957284
1	13.116828	-4.553358	-1.055449
47	0.000000	0.000000	0.000000

Table S6. Atom coordinates of ^{3}[dmaphPc]$^+$ in D_4 symmetry (in Å).

6	1.135501	2.820903	0.000097
7	2.407708	2.407708	0.000000
7	0.000000	2.052650	0.000000
6	-1.135501	2.820903	-0.000097
7	-2.407708	2.407708	-0.000000
6	-2.820903	1.135501	0.000097
7	-2.052650	0.000000	0.000000
6	-2.820903	-1.135501	-0.000097
6	0.707214	4.222672	0.002485
6	-0.707214	4.222672	-0.002485
6	-4.222672	0.707214	0.002485
6	-4.222672	-0.707214	-0.002485
7	-2.407708	-2.407708	0.000000
6	-1.135501	-2.820903	0.000097
7	-0.000000	-2.052650	0.000000
6	1.135501	-2.820903	-0.000097
6	-0.707214	-4.222672	0.002485
6	0.707214	-4.222672	-0.002485
7	2.407708	-2.407708	-0.000000
6	2.820903	-1.135501	0.000097
7	2.052650	-0.000000	0.000000
6	2.820903	1.135501	-0.000097
6	4.222672	-0.707214	0.002485
6	4.222672	0.707214	-0.002485
6	1.413000	5.417876	-0.006532
6	0.716362	6.646496	-0.016064
6	-0.716362	6.646496	0.016064
6	-1.413000	5.417876	0.006532
6	1.519649	7.890666	-0.105572
6	2.653258	8.080791	0.704382
6	3.436372	9.227761	0.620613
6	3.125362	10.256245	-0.304792
6	1.993367	10.054254	-1.138110
6	1.216131	8.909416	-1.028265
6	-1.519649	7.890666	0.105572
6	-2.653258	8.080791	-0.704382
6	-3.436372	9.227761	-0.620613
6	-3.125362	10.256245	0.304792
6	-1.993367	10.054254	1.138110
6	-1.216131	8.909416	1.028265
6	-5.417876	1.413000	-0.006532
6	-6.646496	0.716362	-0.016064
6	-6.646496	-0.716362	0.016064
6	-5.417876	-1.413000	0.006532
6	-7.890666	1.519649	-0.105572
6	-8.080791	2.653258	0.704382
6	-9.227761	3.436372	0.620613
6	-10.256245	3.125362	-0.304792
6	-10.054254	1.993367	-1.138110

6	-8.909416	1.216131	-1.028265
6	-7.890666	-1.519649	0.105572
6	-8.909416	-1.216131	1.028265
6	-10.054254	-1.993367	1.138110
6	-10.256245	-3.125362	0.304792
6	-9.227761	-3.436372	-0.620613
6	-8.080791	-2.653258	-0.704382
6	-1.413000	-5.417876	-0.006532
6	-0.716362	-6.646496	-0.016064
6	0.716362	-6.646496	0.016064
6	1.413000	-5.417876	0.006532
6	-1.519649	-7.890666	-0.105572
6	-2.653258	-8.080791	0.704382
6	-3.436372	-9.227761	0.620613
6	-3.125362	-10.256245	-0.304792
6	-1.993367	-10.054254	-1.138110
6	-1.216131	-8.909416	-1.028265
6	1.519649	-7.890666	0.105572
6	2.653258	-8.080791	-0.704382
6	3.436372	-9.227761	-0.620613
6	3.125362	-10.256245	0.304792
6	1.993367	-10.054254	1.138110
6	1.216131	-8.909416	1.028265
6	5.417876	-1.413000	-0.006532
6	6.646496	-0.716362	-0.016064
6	6.646496	0.716362	0.016064
6	5.417876	1.413000	0.006532
6	7.890666	-1.519649	-0.105572
6	8.909416	-1.216131	-1.028265
6	10.054254	-1.993367	-1.138110
6	10.256245	-3.125362	-0.304792
6	9.227761	-3.436372	0.620613
6	8.080791	-2.653258	0.704382
6	7.890666	1.519649	0.105572
6	8.080791	2.653258	-0.704382
6	9.227761	3.436372	-0.620613
6	10.256245	3.125362	0.304792
6	10.054254	1.993367	1.138110
6	8.909416	1.216131	1.028265
1	2.502818	5.405130	-0.049007
1	-2.502818	5.405130	0.049007
1	2.918777	7.319722	1.442261
1	4.291855	9.321449	1.287380
7	3.887015	11.403379	-0.391484
1	1.714694	10.797989	-1.882621
1	0.351941	8.797959	-1.684621
1	-2.918777	7.319722	-1.442261
1	-4.291855	9.321449	-1.287380
7	-3.887015	11.403379	0.391484
1	-1.714694	10.797989	1.882621
1	-0.351941	8.797959	1.684621

1	-5.405130	2.502818	-0.049007
1	-5.405130	-2.502818	0.049007
1	-7.319722	2.918777	1.442261
1	-9.321449	4.291855	1.287380
7	-11.403379	3.887015	-0.391484
1	-10.797989	1.714694	-1.882621
1	-8.797959	0.351941	-1.684621
1	-8.797959	-0.351941	1.684621
1	-10.797989	-1.714694	1.882621
7	-11.403379	-3.887015	0.391484
1	-9.321449	-4.291855	-1.287380
1	-7.319722	-2.918777	-1.442261
1	-2.502818	-5.405130	-0.049007
1	2.502818	-5.405130	0.049007
1	-2.918777	-7.319722	1.442261
1	-4.291855	-9.321449	1.287380
7	-3.887015	-11.403379	-0.391484
1	-1.714694	-10.797989	-1.882621
1	-0.351941	-8.797959	-1.684621
1	2.918777	-7.319722	-1.442261
1	4.291855	-9.321449	-1.287380
7	3.887015	-11.403379	0.391484
1	1.714694	-10.797989	1.882621
1	0.351941	-8.797959	1.684621
1	5.405130	-2.502818	-0.049007
1	5.405130	2.502818	0.049007
1	8.797959	-0.351941	-1.684621
1	10.797989	-1.714694	-1.882621
7	11.403379	-3.887015	-0.391484
1	9.321449	-4.291855	1.287380
1	7.319722	-2.918777	1.442261
1	7.319722	2.918777	-1.442261
1	9.321449	4.291855	-1.287380
7	11.403379	3.887015	0.391484
1	10.797989	1.714694	1.882621
1	8.797959	0.351941	1.684621
6	-3.582242	-12.404954	-1.397396
6	-5.081471	-11.544807	0.420922
6	5.081471	-11.544807	-0.420922
6	3.582242	-12.404954	1.397396
6	12.404954	-3.582242	-1.397396
6	11.544807	-5.081471	0.420922
6	12.404954	3.582242	1.397396
6	11.544807	5.081471	-0.420922
6	5.081471	11.544807	0.420922
6	3.582242	12.404954	-1.397396
6	-3.582242	12.404954	1.397396
6	-5.081471	11.544807	-0.420922
6	-11.544807	5.081471	0.420922
6	-12.404954	3.582242	-1.397396
6	-12.404954	-3.582242	1.397396

6	-11.544807	-5.081471	-0.420922
1	-2.561011	-12.807403	-1.274458
1	-3.665583	-12.005335	-2.425880
1	-4.284747	-13.242003	-1.300727
1	-5.546506	-12.517331	0.217397
1	-5.828492	-10.756669	0.207696
1	-4.847643	-11.503329	1.499825
1	5.828492	-10.756669	-0.207696
1	4.847643	-11.503329	-1.499825
1	5.546506	-12.517331	-0.217397
1	4.284747	-13.242003	1.300727
1	2.561011	-12.807403	1.274458
1	3.665583	-12.005335	2.425880
1	12.807403	-2.561011	-1.274458
1	12.005335	-3.665583	-2.425880
1	13.242003	-4.284747	-1.300727
1	10.756669	-5.828492	0.207696
1	11.503329	-4.847643	1.499825
1	12.517331	-5.546506	0.217397
1	12.807403	2.561011	1.274458
1	12.005335	3.665583	2.425880
1	13.242003	4.284747	1.300727
1	10.756669	5.828492	-0.207696
1	11.503329	4.847643	-1.499825
1	12.517331	5.546506	-0.217397
1	5.828492	10.756669	0.207696
1	4.847643	11.503329	1.499825
1	5.546506	12.517331	0.217397
1	2.561011	12.807403	-1.274458
1	3.665583	12.005335	-2.425880
1	4.284747	13.242003	-1.300727
1	-2.561011	12.807403	1.274458
1	-3.665583	12.005335	2.425880
1	-4.284747	13.242003	1.300727
1	-5.828492	10.756669	-0.207696
1	-4.847643	11.503329	-1.499825
1	-5.546506	12.517331	-0.217397
1	-10.756669	5.828492	0.207696
1	-11.503329	4.847643	1.499825
1	-12.517331	5.546506	0.217397
1	-12.807403	2.561011	-1.274458
1	-12.005335	3.665583	-2.425880
1	-13.242003	4.284747	-1.300727
1	-12.807403	-2.561011	1.274458
1	-12.005335	-3.665583	2.425880
1	-13.242003	-4.284747	1.300727
1	-10.756669	-5.828492	-0.207696
1	-11.503329	-4.847643	-1.499825
1	-12.517331	-5.546506	-0.217397
47	0.000000	0.000000	0.000000

Table S7. Atom coordinates of [2][dmaphPc][0] in D_{4h} symmetry (in Å).

6	-1.135893	2.821352	0.000000
7	-2.406560	2.406560	0.000000
7	0.000000	2.056524	0.000000
6	1.135893	2.821352	0.000000
7	2.406560	2.406560	0.000000
6	2.821352	1.135893	0.000000
7	2.056524	-0.000000	0.000000
6	2.821352	-1.135893	-0.000000
6	-0.707932	4.224197	0.000000
6	0.707932	4.224197	0.000000
6	4.224197	0.707932	0.000000
6	4.224197	-0.707932	-0.000000
7	2.406560	-2.406560	0.000000
6	1.135893	-2.821352	0.000000
7	-0.000000	-2.056524	0.000000
6	-1.135893	-2.821352	-0.000000
6	0.707932	-4.224197	0.000000
6	-0.707932	-4.224197	-0.000000
7	-2.406560	-2.406560	-0.000000
6	-2.821352	-1.135893	0.000000
7	-2.056524	0.000000	0.000000
6	-2.821352	1.135893	-0.000000
6	-4.224197	-0.707932	0.000000
6	-4.224197	0.707932	-0.000000
6	-1.419377	5.422863	0.000000
6	-0.712396	6.634755	0.000000
6	0.712396	6.634755	0.000000
6	1.419377	5.422863	0.000000
6	-1.465160	7.925713	0.000000
6	-1.832305	8.554251	1.196795
6	-2.530400	9.760616	1.207109
6	-2.886010	10.407972	0.000000
6	-2.530400	9.760616	-1.207109
6	-1.832305	8.554251	-1.196795
6	1.465160	7.925713	0.000000
6	1.832305	8.554251	-1.196795
6	2.530400	9.760616	-1.207109
6	2.886010	10.407972	0.000000
6	2.530400	9.760616	1.207109
6	1.832305	8.554251	1.196795
6	5.422863	1.419377	0.000000
6	6.634755	0.712396	0.000000
6	6.634755	-0.712396	-0.000000
6	5.422863	-1.419377	-0.000000
6	7.925713	1.465160	0.000000
6	8.554251	1.832305	1.196795
6	9.760616	2.530400	1.207109
6	10.407972	2.886010	0.000000
6	9.760616	2.530400	-1.207109

6	8.554251	1.832305	-1.196795
6	7.925713	-1.465160	-0.000000
6	8.554251	-1.832305	1.196795
6	9.760616	-2.530400	1.207109
6	10.407972	-2.886010	-0.000000
6	9.760616	-2.530400	-1.207109
6	8.554251	-1.832305	-1.196795
6	1.419377	-5.422863	0.000000
6	0.712396	-6.634755	0.000000
6	-0.712396	-6.634755	-0.000000
6	-1.419377	-5.422863	-0.000000
6	1.465160	-7.925713	0.000000
6	1.832305	-8.554251	1.196795
6	2.530400	-9.760616	1.207109
6	2.886010	-10.407972	0.000000
6	2.530400	-9.760616	-1.207109
6	1.832305	-8.554251	-1.196795
6	-1.465160	-7.925713	-0.000000
6	-1.832305	-8.554251	-1.196795
6	-2.530400	-9.760616	-1.207109
6	-2.886010	-10.407972	-0.000000
6	-2.530400	-9.760616	1.207109
6	-1.832305	-8.554251	1.196795
6	-5.422863	-1.419377	0.000000
6	-6.634755	-0.712396	0.000000
6	-6.634755	0.712396	-0.000000
6	-5.422863	1.419377	-0.000000
6	-7.925713	-1.465160	0.000000
6	-8.554251	-1.832305	-1.196795
6	-9.760616	-2.530400	-1.207109
6	-10.407972	-2.886010	0.000000
6	-9.760616	-2.530400	1.207109
6	-8.554251	-1.832305	1.196795
6	-7.925713	1.465160	-0.000000
6	-8.554251	1.832305	-1.196795
6	-9.760616	2.530400	-1.207109
6	-10.407972	2.886010	-0.000000
6	-9.760616	2.530400	1.207109
6	-8.554251	1.832305	1.196795
1	-2.510415	5.418042	0.000000
1	2.510415	5.418042	0.000000
1	-1.566258	8.087569	2.148142
1	-2.793204	10.197800	2.169087
7	-3.550731	11.633064	0.000000
1	-2.793204	10.197800	-2.169087
1	-1.566258	8.087569	-2.148142
1	1.566258	8.087569	-2.148142
1	2.793204	10.197800	-2.169087
7	3.550731	11.633064	0.000000
1	2.793204	10.197800	2.169087
1	1.566258	8.087569	2.148142

1	5.418042	2.510415	0.000000
1	5.418042	-2.510415	-0.000000
1	8.087569	1.566258	2.148142
1	10.197800	2.793204	2.169087
7	11.633064	3.550731	0.000000
1	10.197800	2.793204	-2.169087
1	8.087569	1.566258	-2.148142
1	8.087569	-1.566258	2.148142
1	10.197800	-2.793204	2.169087
7	11.633064	-3.550731	-0.000000
1	10.197800	-2.793204	-2.169087
1	8.087569	-1.566258	-2.148142
1	2.510415	-5.418042	0.000000
1	-2.510415	-5.418042	-0.000000
1	1.566258	-8.087569	2.148142
1	2.793204	-10.197800	2.169087
7	3.550731	-11.633064	0.000000
1	2.793204	-10.197800	-2.169087
1	1.566258	-8.087569	-2.148142
1	-1.566258	-8.087569	-2.148142
1	-2.793204	-10.197800	-2.169087
7	-3.550731	-11.633064	-0.000000
1	-2.793204	-10.197800	2.169087
1	-1.566258	-8.087569	2.148142
1	-5.418042	-2.510415	0.000000
1	-5.418042	2.510415	-0.000000
1	-8.087569	-1.566258	-2.148142
1	-10.197800	-2.793204	-2.169087
7	-11.633064	-3.550731	0.000000
1	-10.197800	-2.793204	2.169087
1	-8.087569	-1.566258	2.148142
1	-8.087569	1.566258	-2.148142
1	-10.197800	2.793204	-2.169087
7	-11.633064	3.550731	-0.000000
1	-10.197800	2.793204	2.169087
1	-8.087569	1.566258	2.148142
6	4.123808	-12.119128	-1.241378
6	4.123808	-12.119128	1.241378
6	-4.123808	-12.119128	-1.241378
6	-4.123808	-12.119128	1.241378
6	-12.119128	-4.123808	-1.241378
6	-12.119128	-4.123808	1.241378
6	-12.119128	4.123808	1.241378
6	-12.119128	4.123808	-1.241378
6	-4.123808	12.119128	1.241378
6	-4.123808	12.119128	-1.241378
6	4.123808	12.119128	1.241378
6	4.123808	12.119128	-1.241378
6	12.119128	4.123808	1.241378
6	12.119128	4.123808	-1.241378
6	12.119128	-4.123808	1.241378

6	12.119128	-4.123808	-1.241378
1	3.343484	-12.275119	-2.004656
1	4.881196	-11.430630	-1.670035
1	4.604003	-13.090481	-1.062375
1	4.604003	-13.090481	1.062375
1	4.881196	-11.430630	1.670035
1	3.343484	-12.275119	2.004656
1	-4.881196	-11.430630	-1.670035
1	-3.343484	-12.275119	-2.004656
1	-4.604003	-13.090481	-1.062375
1	-4.604003	-13.090481	1.062375
1	-3.343484	-12.275119	2.004656
1	-4.881196	-11.430630	1.670035
1	-12.275119	-3.343484	-2.004656
1	-11.430630	-4.881196	-1.670035
1	-13.090481	-4.604003	-1.062375
1	-11.430630	-4.881196	1.670035
1	-12.275119	-3.343484	2.004656
1	-13.090481	-4.604003	1.062375
1	-12.275119	3.343484	2.004656
1	-11.430630	4.881196	1.670035
1	-13.090481	4.604003	1.062375
1	-11.430630	4.881196	-1.670035
1	-12.275119	3.343484	-2.004656
1	-13.090481	4.604003	-1.062375
1	-4.881196	11.430630	1.670035
1	-3.343484	12.275119	2.004656
1	-4.604003	13.090481	1.062375
1	-3.343484	12.275119	-2.004656
1	-4.881196	11.430630	-1.670035
1	-4.604003	13.090481	-1.062375
1	3.343484	12.275119	2.004656
1	4.881196	11.430630	1.670035
1	4.604003	13.090481	1.062375
1	4.881196	11.430630	-1.670035
1	3.343484	12.275119	-2.004656
1	4.604003	13.090481	-1.062375
1	11.430630	4.881196	1.670035
1	12.275119	3.343484	2.004656
1	13.090481	4.604003	1.062375
1	12.275119	3.343484	-2.004656
1	11.430630	4.881196	-1.670035
1	13.090481	4.604003	-1.062375
1	12.275119	-3.343484	2.004656
1	11.430630	-4.881196	1.670035
1	13.090481	-4.604003	1.062375
1	11.430630	-4.881196	-1.670035
1	12.275119	-3.343484	-2.004656
1	13.090481	-4.604003	-1.062375
47	0.000000	0.000000	0.000000

Table S8. Atom coordinates of ^{2}[dmaphPc]^{0} in D_4 symmetry (in Å).

C	-1.136332194096	-2.821863041952	-0.000004128210
N	-2.407311814908	-2.407311814908	-0.000000000000
N	-0.000000000000	-2.056683061226	-0.000000000000
C	1.136332194096	-2.821863041952	0.000004128210
N	2.407311814908	-2.407311814908	-0.000000000000
C	2.821863041952	-1.136332194096	-0.000004128210
N	2.056683061226	-0.000000000000	-0.000000000000
C	2.821863041952	1.136332194096	0.000004128210
C	-0.707602490742	-4.223334397970	0.001598002519
C	0.707602490742	-4.223334397970	-0.001598002519
C	4.223334397970	-0.707602490742	0.001598002519
C	4.223334397970	0.707602490742	-0.001598002519
N	2.407311814908	2.407311814908	-0.000000000000
C	1.136332194096	2.821863041952	-0.000004128210
N	0.000000000000	2.056683061226	-0.000000000000
C	-1.136332194096	2.821863041952	0.000004128210
C	0.707602490742	4.223334397970	0.001598002519
C	-0.707602490742	4.223334397970	-0.001598002519
N	-2.407311814908	2.407311814908	-0.000000000000
C	-2.821863041952	1.136332194096	-0.000004128210
N	-2.056683061226	0.000000000000	-0.000000000000
C	-2.821863041952	-1.136332194096	0.000004128210
C	-4.223334397970	0.707602490742	0.001598002519
C	-4.223334397970	-0.707602490742	-0.001598002519
C	-1.413445037028	-5.422497958501	-0.007911159989
C	-0.716571053315	-6.644325699358	-0.013548552025
C	0.716571053315	-6.644325699358	0.013548552025
C	1.413445037028	-5.422497958501	0.007911159989
C	-1.513452517103	-7.899140519311	-0.082676459503
C	-2.609868727294	-8.107377219426	0.769338115662
C	-3.387434701877	-9.262188512655	0.704591195533
C	-3.098952556743	-10.283492071089	-0.231206575961
C	-2.009680426696	-10.061163089945	-1.109449675702
C	-1.240215635895	-8.905931317822	-1.024549203641
C	1.513452517103	-7.899140519311	0.082676459503
C	2.609868727294	-8.107377219426	-0.769338115662
C	3.387434701877	-9.262188512655	-0.704591195533
C	3.098952556742	-10.283492071089	0.231206575961
C	2.009680426696	-10.061163089945	1.109449675702
C	1.240215635895	-8.905931317822	1.024549203641
C	5.422497958501	-1.413445037028	-0.007911159989
C	6.644325699358	-0.716571053315	-0.013548552025
C	6.644325699358	0.716571053315	0.013548552025
C	5.422497958501	1.413445037028	0.007911159989
C	7.899140519311	-1.513452517103	-0.082676459503
C	8.107377219426	-2.609868727294	0.769338115662
C	9.262188512655	-3.387434701877	0.704591195533
C	10.283492071089	-3.098952556743	-0.231206575961
C	10.061163089945	-2.009680426696	-1.109449675702

C	8.905931317822	-1.240215635895	-1.024549203641
C	7.899140519311	1.513452517103	0.082676459503
C	8.905931317822	1.240215635895	1.024549203641
C	10.061163089945	2.009680426696	1.109449675702
C	10.283492071089	3.098952556742	0.231206575961
C	9.262188512655	3.387434701877	-0.704591195533
C	8.107377219426	2.609868727294	-0.769338115662
C	1.413445037028	5.422497958501	-0.007911159989
C	0.716571053315	6.644325699358	-0.013548552025
C	-0.716571053315	6.644325699358	0.013548552025
C	-1.413445037027	5.422497958501	0.007911159989
C	1.513452517103	7.899140519311	-0.082676459503
C	2.609868727294	8.107377219426	0.769338115662
C	3.387434701877	9.262188512655	0.704591195533
C	3.098952556743	10.283492071089	-0.231206575961
C	2.009680426696	10.061163089945	-1.109449675702
C	1.240215635895	8.905931317822	-1.024549203641
C	-1.513452517103	7.899140519311	0.082676459503
C	-2.609868727294	8.107377219426	-0.769338115662
C	-3.387434701877	9.262188512655	-0.704591195533
C	-3.098952556742	10.283492071089	0.231206575961
C	-2.009680426696	10.061163089945	1.109449675702
C	-1.240215635895	8.905931317822	1.024549203641
C	-5.422497958501	1.413445037028	-0.007911159989
C	-6.644325699358	0.716571053315	-0.013548552025
C	-6.644325699358	-0.716571053315	0.013548552025
C	-5.422497958501	-1.413445037027	0.007911159989
C	-7.899140519311	1.513452517103	-0.082676459503
C	-8.905931317822	1.240215635895	-1.024549203641
C	-10.061163089945	2.009680426696	-1.109449675702
C	-10.283492071089	3.098952556743	-0.231206575961
C	-9.262188512655	3.387434701877	0.704591195533
C	-8.107377219426	2.609868727294	0.769338115662
C	-7.899140519311	-1.513452517103	0.082676459503
C	-8.107377219426	-2.609868727294	-0.769338115662
C	-9.262188512655	-3.387434701877	-0.704591195533
C	-10.283492071089	-3.098952556742	0.231206575961
C	-10.061163089945	-2.009680426696	1.109449675702
C	-8.905931317822	-1.240215635895	1.024549203641
H	-2.503675871718	-5.410434153676	-0.046761799361
H	2.503675871718	-5.410434153676	0.046761799361
H	-2.855984660293	-7.349855942901	1.517449410535
H	-4.220782276242	-9.363665437458	1.398038330343
N	-3.846158401527	-11.456834990640	-0.282031217249
H	-1.753217251075	-10.795723144587	-1.871095775358
H	-0.405522769942	-8.778794819140	-1.715654480974
H	2.855984660293	-7.349855942901	-1.517449410535
H	4.220782276242	-9.363665437458	-1.398038330343
N	3.846158401527	-11.456834990640	0.282031217249
H	1.753217251075	-10.795723144587	1.871095775358
H	0.405522769942	-8.778794819140	1.715654480974

H	5.410434153676	-2.503675871718	-0.046761799361
H	5.410434153676	2.503675871718	0.046761799361
H	7.349855942901	-2.855984660293	1.517449410535
H	9.363665437458	-4.220782276242	1.398038330343
N	11.456834990640	-3.846158401527	-0.282031217249
H	10.795723144587	-1.753217251075	-1.871095775358
H	8.778794819140	-0.405522769942	-1.715654480974
H	8.778794819140	0.405522769942	1.715654480974
H	10.795723144587	1.753217251075	1.871095775358
N	11.456834990640	3.846158401527	0.282031217249
H	9.363665437458	4.220782276242	-1.398038330343
H	7.349855942901	2.855984660293	-1.517449410535
H	2.503675871718	5.410434153676	-0.046761799361
H	-2.503675871718	5.410434153676	0.046761799361
H	2.855984660293	7.349855942901	1.517449410535
H	4.220782276242	9.363665437458	1.398038330343
N	3.846158401527	11.456834990640	-0.282031217249
H	1.753217251075	10.795723144587	-1.871095775358
H	0.405522769942	8.778794819140	-1.715654480974
H	-2.855984660293	7.349855942901	-1.517449410535
H	-4.220782276242	9.363665437458	-1.398038330343
N	-3.846158401527	11.456834990640	0.282031217249
H	-1.753217251075	10.795723144587	1.871095775358
H	-0.405522769942	8.778794819140	1.715654480974
H	-5.410434153676	2.503675871718	-0.046761799361
H	-5.410434153676	-2.503675871718	0.046761799361
H	-8.778794819140	0.405522769942	-1.715654480974
H	-10.795723144587	1.753217251075	-1.871095775358
N	-11.456834990640	3.846158401527	-0.282031217249
H	-9.363665437458	4.220782276242	1.398038330343
H	-7.349855942901	2.855984660293	1.517449410535
H	-7.349855942901	-2.855984660293	-1.517449410535
H	-9.363665437458	-4.220782276242	-1.398038330343
N	-11.456834990640	-3.846158401527	0.282031217249
H	-10.795723144587	-1.753217251075	1.871095775358
H	-8.778794819140	-0.405522769942	1.715654480974
C	3.673773783914	12.352914776177	-1.410689939554
C	5.097376311454	11.525199361867	0.450123924542
C	-5.097376311453	11.525199361867	-0.450123924542
C	-3.673773783914	12.352914776177	1.410689939554
C	-12.352914776177	3.673773783914	-1.410689939554
C	-11.525199361867	5.097376311454	0.450123924542
C	-12.352914776177	-3.673773783914	1.410689939554
C	-11.525199361867	-5.097376311453	-0.450123924542
C	-5.097376311454	-11.525199361867	0.450123924542
C	-3.673773783914	-12.352914776177	-1.410689939554
C	3.673773783914	-12.352914776177	1.410689939554
C	5.097376311453	-11.525199361867	-0.450123924542
C	11.525199361867	-5.097376311453	0.450123924542
C	12.352914776177	-3.673773783914	-1.410689939554
C	12.352914776177	3.673773783914	1.410689939554

C	11.525199361867	5.097376311453	-0.450123924542
H	2.636856116103	12.723122683296	-1.468769547338
H	3.916801838147	11.877966742521	-2.383366083151
H	4.326502678651	13.226596564167	-1.281817729821
H	5.549709980699	12.514881194675	0.302347807810
H	5.829975809731	10.756328952125	0.129419783223
H	4.931727985701	11.398166232978	1.532908387442
H	-5.829975809731	10.756328952125	-0.129419783223
H	-4.931727985701	11.398166232978	-1.532908387442
H	-5.549709980699	12.514881194675	-0.302347807810
H	-4.326502678650	13.226596564167	1.281817729821
H	-2.636856116103	12.723122683296	1.468769547338
H	-3.916801838147	11.877966742521	2.383366083151
H	-12.723122683296	2.636856116103	-1.468769547338
H	-11.877966742521	3.916801838147	-2.383366083151
H	-13.226596564167	4.326502678651	-1.281817729821
H	-10.756328952125	5.829975809731	0.129419783223
H	-11.398166232978	4.931727985701	1.532908387442
H	-12.514881194675	5.549709980699	0.302347807810
H	-12.723122683296	-2.636856116103	1.468769547338
H	-11.877966742521	-3.916801838147	2.383366083151
H	-13.226596564167	-4.326502678650	1.281817729821
H	-10.756328952125	-5.829975809731	-0.129419783223
H	-11.398166232978	-4.931727985701	-1.532908387442
H	-12.514881194675	-5.549709980699	-0.302347807810
H	-5.829975809731	-10.756328952125	0.129419783223
H	-4.931727985701	-11.398166232978	1.532908387442
H	-5.549709980699	-12.514881194675	0.302347807810
H	-2.636856116103	-12.723122683296	-1.468769547338
H	-3.916801838147	-11.877966742521	-2.383366083151
H	-4.326502678651	-13.226596564167	-1.281817729821
H	2.636856116103	-12.723122683296	1.468769547338
H	3.916801838147	-11.877966742521	2.383366083151
H	4.326502678650	-13.226596564167	1.281817729821
H	5.829975809731	-10.756328952125	-0.129419783223
H	4.931727985701	-11.398166232978	-1.532908387442
H	5.549709980699	-12.514881194675	-0.302347807810
H	10.756328952125	-5.829975809731	0.129419783223
H	11.398166232978	-4.931727985701	1.532908387442
H	12.514881194675	-5.549709980699	0.302347807810
H	12.723122683296	-2.636856116103	-1.468769547338
H	11.877966742521	-3.916801838147	-2.383366083151
H	13.226596564167	-4.326502678651	-1.281817729821
H	12.723122683296	2.636856116103	1.468769547338
H	11.877966742521	3.916801838147	2.383366083151
H	13.226596564167	4.326502678650	1.281817729821
H	10.756328952125	5.829975809731	-0.129419783223
H	11.398166232978	4.931727985701	-1.532908387442
H	12.514881194675	5.549709980699	-0.302347807810
Ag	0.000000000000	0.000000000000	-0.000000000000

Table S9. Atom coordinates of [2][dmaphPc][0] in D_2 symmetry (in Å).

6	1.135229	-2.826579	-0.000157
7	2.430915	-2.404105	-0.000293
7	-0.000000	-2.059532	0.000000
6	-1.135229	-2.826579	0.000157
7	-2.430915	-2.404105	0.000293
6	-2.822654	-1.143391	0.000352
7	-2.054826	-0.000000	0.000000
6	-2.822654	1.143391	-0.000352
6	0.711182	-4.216301	-0.001659
6	-0.711182	-4.216301	0.001659
6	-4.235186	-0.704742	-0.001475
6	-4.235186	0.704742	0.001475
7	-2.430915	2.404105	-0.000293
6	-1.135229	2.826579	-0.000157
7	-0.000000	2.059532	0.000000
6	1.135229	2.826579	0.000157
6	-0.711182	4.216301	-0.001659
6	0.711182	4.216301	0.001659
7	2.430915	2.404105	0.000293
6	2.822654	1.143391	0.000352
7	2.054826	0.000000	0.000000
6	2.822654	-1.143391	-0.000352
6	4.235186	0.704742	-0.001475
6	4.235186	-0.704742	0.001475
6	1.413930	-5.419323	0.007898
6	0.717427	-6.641529	0.013979
6	-0.717427	-6.641529	-0.013979
6	-1.413930	-5.419323	-0.007898
6	1.514881	-7.894868	0.090171
6	2.620845	-8.102001	-0.750227
6	3.399464	-9.255624	-0.677601
6	3.102460	-10.277941	0.254312
6	2.003469	-10.057556	1.120821
6	1.233500	-8.903297	1.028450
6	-1.514881	-7.894868	-0.090171
6	-2.620845	-8.102001	0.750227
6	-3.399464	-9.255624	0.677601
6	-3.102460	-10.277941	-0.254312
6	-2.003469	-10.057556	-1.120821
6	-1.233500	-8.903297	-1.028450
6	-5.427263	-1.410993	0.007502
6	-6.656560	-0.713283	0.013828
6	-6.656560	0.713283	-0.013828
6	-5.427263	1.410993	-0.007502
6	-7.906908	-1.515532	0.086638
6	-8.106382	-2.621477	-0.755507
6	-9.257461	-3.403744	-0.687512
6	-10.282883	-3.112191	0.243215
6	-10.068690	-2.013712	1.112477

6	-8.917653	-1.238941	1.023569
6	-7.906908	1.515532	-0.086638
6	-8.917653	1.238941	-1.023569
6	-10.068690	2.013712	-1.112477
6	-10.282883	3.112191	-0.243215
6	-9.257461	3.403744	0.687512
6	-8.106382	2.621477	0.755507
6	-1.413930	5.419323	0.007898
6	-0.717427	6.641529	0.013979
6	0.717427	6.641529	-0.013979
6	1.413930	5.419323	-0.007898
6	-1.514881	7.894868	0.090171
6	-2.620845	8.102001	-0.750227
6	-3.399464	9.255624	-0.677601
6	-3.102460	10.277941	0.254312
6	-2.003469	10.057556	1.120821
6	-1.233500	8.903297	1.028450
6	1.514881	7.894868	-0.090171
6	2.620845	8.102001	0.750227
6	3.399464	9.255624	0.677601
6	3.102460	10.277941	-0.254312
6	2.003469	10.057556	-1.120821
6	1.233500	8.903297	-1.028450
6	5.427263	1.410993	0.007502
6	6.656560	0.713283	0.013828
6	6.656560	-0.713283	-0.013828
6	5.427263	-1.410993	-0.007502
6	7.906908	1.515532	0.086638
6	8.917653	1.238941	1.023569
6	10.068690	2.013712	1.112477
6	10.282883	3.112191	0.243215
6	9.257461	3.403744	-0.687512
6	8.106382	2.621477	-0.755507
6	7.906908	-1.515532	-0.086638
6	8.106382	-2.621477	0.755507
6	9.257461	-3.403744	0.687512
6	10.282883	-3.112191	-0.243215
6	10.068690	-2.013712	-1.112477
6	8.917653	-1.238941	-1.023569
1	2.504263	-5.408465	0.048175
1	-2.504263	-5.408465	-0.048175
1	2.873616	-7.344184	-1.495794
1	4.240168	-9.355617	-1.362393
7	3.850796	-11.450700	0.312402
1	1.739950	-10.792789	1.879455
1	0.391778	-8.777586	1.711208
1	-2.873616	-7.344184	1.495794
1	-4.240168	-9.355617	1.362393
7	-3.850796	-11.450700	-0.312402
1	-1.739950	-10.792789	-1.879455
1	-0.391778	-8.777586	-1.711208

1	-5.412401	-2.501226	0.045034
1	-5.412401	2.501226	-0.045034
1	-7.345531	-2.871505	-1.498910
1	-9.352571	-4.243964	-1.373459
7	-11.450922	-3.865413	0.298447
1	-10.806187	-1.755048	1.870495
1	-8.796406	-0.397792	1.707816
1	-8.796406	0.397792	-1.707816
1	-10.806187	1.755048	-1.870495
7	-11.450922	3.865413	-0.298447
1	-9.352571	4.243964	1.373459
1	-7.345531	2.871505	1.498910
1	-2.504263	5.408465	0.048175
1	2.504263	5.408465	-0.048175
1	-2.873616	7.344184	-1.495794
1	-4.240168	9.355617	-1.362393
7	-3.850796	11.450700	0.312402
1	-1.739950	10.792789	1.879455
1	-0.391778	8.777586	1.711208
1	2.873616	7.344184	1.495794
1	4.240168	9.355617	1.362393
7	3.850796	11.450700	-0.312402
1	1.739950	10.792789	-1.879455
1	0.391778	8.777586	-1.711208
1	5.412401	2.501226	0.045034
1	5.412401	-2.501226	-0.045034
1	8.796406	0.397792	1.707816
1	10.806187	1.755048	1.870495
7	11.450922	3.865413	0.298447
1	9.352571	4.243964	-1.373459
1	7.345531	2.871505	-1.498910
1	7.345531	-2.871505	1.498910
1	9.352571	-4.243964	1.373459
7	11.450922	-3.865413	-0.298447
1	10.806187	-1.755048	-1.870495
1	8.796406	-0.397792	-1.707816
6	-3.668944	12.346173	1.440016
6	-5.111119	11.515441	-0.404180
6	5.111119	11.515441	0.404180
6	3.668944	12.346173	-1.440016
6	12.355961	3.683308	1.418457
6	11.515008	5.119944	-0.428581
6	12.355961	-3.683308	-1.418457
6	11.515008	-5.119944	0.428581
6	5.111119	-11.515441	-0.404180
6	3.668944	-12.346173	1.440016
6	-3.668944	-12.346173	-1.440016
6	-5.111119	-11.515441	0.404180
6	-11.515008	-5.119944	-0.428581
6	-12.355961	-3.683308	1.418457
6	-12.355961	3.683308	-1.418457

6	-11.515008	5.119944	0.428581
1	-2.631797	12.716938	1.488924
1	-3.902828	11.870548	2.414668
1	-4.323309	13.219584	1.317458
1	-5.563374	12.504633	-0.252683
1	-5.838342	10.745774	-0.073076
1	-4.958653	11.386648	-1.488664
1	5.838342	10.745774	0.073076
1	4.958653	11.386648	1.488664
1	5.563374	12.504633	0.252683
1	4.323309	13.219584	-1.317458
1	2.631797	12.716938	-1.488924
1	3.902828	11.870548	-2.414668
1	12.732550	2.647846	1.460508
1	11.886040	3.910917	2.397098
1	13.224746	4.342960	1.292404
1	10.742692	5.847475	-0.105285
1	11.389766	4.958462	-1.512265
1	12.502417	5.575947	-0.277500
1	12.732550	-2.647846	-1.460508
1	11.886040	-3.910917	-2.397098
1	13.224746	-4.342960	-1.292404
1	10.742692	-5.847475	0.105285
1	11.389766	-4.958462	1.512265
1	12.502417	-5.575947	0.277500
1	5.838342	-10.745774	-0.073076
1	4.958653	-11.386648	-1.488664
1	5.563374	-12.504633	-0.252683
1	2.631797	-12.716938	1.488924
1	3.902828	-11.870548	2.414668
1	4.323309	-13.219584	1.317458
1	-2.631797	-12.716938	-1.488924
1	-3.902828	-11.870548	-2.414668
1	-4.323309	-13.219584	-1.317458
1	-5.838342	-10.745774	0.073076
1	-4.958653	-11.386648	1.488664
1	-5.563374	-12.504633	0.252683
1	-10.742692	-5.847475	-0.105285
1	-11.389766	-4.958462	-1.512265
1	-12.502417	-5.575947	-0.277500
1	-12.732550	-2.647846	1.460508
1	-11.886040	-3.910917	2.397098
1	-13.224746	-4.342960	1.292404
1	-12.732550	2.647846	-1.460508
1	-11.886040	3.910917	-2.397098
1	-13.224746	4.342960	-1.292404
1	-10.742692	5.847475	0.105285
1	-11.389766	4.958462	1.512265
1	-12.502417	5.575947	0.277500
47	-0.000000	0.000000	0.000000

Table S10. Atom coordinates of [1][dmaphPc]$^{-}$ in D_2 symmetry (in Å).

6	1.139235	2.802627	0.002868
7	2.442780	2.399220	0.004213
7	0.000000	2.011982	0.000000
6	-1.139235	2.802627	-0.002868
7	-2.442780	2.399220	-0.004213
6	-2.798306	1.152729	-0.002754
7	-2.003947	0.000000	0.000000
6	-2.798306	-1.152729	0.002754
6	0.715342	4.155803	0.003029
6	-0.715342	4.155803	-0.003029
6	-4.194744	0.701674	-0.001024
6	-4.194744	-0.701674	0.001024
7	-2.442780	-2.399220	0.004213
6	-1.139235	-2.802627	0.002868
7	-0.000000	-2.011982	0.000000
6	1.139235	-2.802627	-0.002868
6	-0.715342	-4.155803	0.003029
6	0.715342	-4.155803	-0.003029
7	2.442780	-2.399220	-0.004213
6	2.798306	-1.152729	-0.002754
7	2.003947	-0.000000	0.000000
6	2.798306	1.152729	0.002754
6	4.194744	-0.701674	-0.001024
6	4.194744	0.701674	0.001024
6	1.418710	5.376332	-0.008484
6	0.727178	6.583187	-0.014060
6	-0.727178	6.583187	0.014060
6	-1.418710	5.376332	0.008484
6	1.514901	7.843307	-0.102016
6	2.626009	8.067239	0.727615
6	3.401285	9.223882	0.636988
6	3.089887	10.239232	-0.294040
6	1.983962	10.009151	-1.144787
6	1.222196	8.848123	-1.041037
6	-1.514901	7.843307	0.102016
6	-2.626009	8.067239	-0.727615
6	-3.401285	9.223882	-0.636988
6	-3.089887	10.239232	0.294040
6	-1.983962	10.009151	1.144787
6	-1.222196	8.848123	1.041037
6	-5.390980	1.411269	-0.013705
6	-6.614293	0.715192	-0.015819
6	-6.614293	-0.715192	0.015819
6	-5.390980	-1.411269	0.013705
6	-7.868995	1.513988	-0.089883
6	-8.076180	2.616609	0.754160
6	-9.228481	3.398836	0.681536
6	-10.250034	3.107016	-0.250338
6	-10.031486	2.011802	-1.119143

6	-8.876682	1.239825	-1.029989
6	-7.868995	-1.513988	0.089883
6	-8.876682	-1.239825	1.029989
6	-10.031486	-2.011802	1.119143
6	-10.250034	-3.107016	0.250338
6	-9.228481	-3.398836	-0.681536
6	-8.076180	-2.616609	-0.754160
6	-1.418710	-5.376332	-0.008484
6	-0.727178	-6.583187	-0.014060
6	0.727178	-6.583187	0.014060
6	1.418710	-5.376332	0.008484
6	-1.514901	-7.843307	-0.102016
6	-2.626009	-8.067239	0.727615
6	-3.401285	-9.223882	0.636988
6	-3.089887	-10.239232	-0.294040
6	-1.983962	-10.009151	-1.144787
6	-1.222196	-8.848123	-1.041037
6	1.514901	-7.843307	0.102016
6	2.626009	-8.067239	-0.727615
6	3.401285	-9.223882	-0.636988
6	3.089887	-10.239232	0.294040
6	1.983962	-10.009151	1.144787
6	1.222196	-8.848123	1.041037
6	5.390980	-1.411269	-0.013705
6	6.614293	-0.715192	-0.015819
6	6.614293	0.715192	0.015819
6	5.390980	1.411269	0.013705
6	7.868995	-1.513988	-0.089883
6	8.876682	-1.239825	-1.029989
6	10.031486	-2.011802	-1.119143
6	10.250034	-3.107016	-0.250338
6	9.228481	-3.398836	0.681536
6	8.076180	-2.616609	0.754160
6	7.868995	1.513988	0.089883
6	8.076180	2.616609	-0.754160
6	9.228481	3.398836	-0.681536
6	10.250034	3.107016	0.250338
6	10.031486	2.011802	1.119143
6	8.876682	1.239825	1.029989
1	2.509277	5.364089	-0.055940
1	-2.509277	5.364089	0.055940
1	2.887905	7.314208	1.474617
1	4.249543	9.329923	1.312230
7	3.834334	11.428283	-0.361868
1	1.705160	10.740225	-1.902600
1	0.375529	8.713806	-1.716129
1	-2.887905	7.314208	-1.474617
1	-4.249543	9.329923	-1.312230
7	-3.834334	11.428283	0.361868
1	-1.705160	10.740225	1.902600
1	-0.375529	8.713806	1.716129

1	-5.371174	2.501326	-0.056487
1	-5.371174	-2.501326	0.056487
1	-7.316110	2.866963	1.497976
1	-9.324709	4.239740	1.366889
7	-11.428840	3.860756	-0.304725
1	-10.766482	1.751292	-1.879343
1	-8.750255	0.401401	-1.716755
1	-8.750255	-0.401401	1.716755
1	-10.766482	-1.751292	1.879343
7	-11.428840	-3.860756	0.304725
1	-9.324709	-4.239740	-1.366889
1	-7.316110	-2.866963	-1.497976
1	-2.509277	-5.364089	-0.055940
1	2.509277	-5.364089	0.055940
1	-2.887905	-7.314208	1.474617
1	-4.249543	-9.329923	1.312230
7	-3.834334	-11.428283	-0.361868
1	-1.705160	-10.740225	-1.902600
1	-0.375529	-8.713806	-1.716129
1	2.887905	-7.314208	-1.474617
1	4.249543	-9.329923	-1.312230
7	3.834334	-11.428283	0.361868
1	1.705160	-10.740225	1.902600
1	0.375529	-8.713806	1.716129
1	5.371174	-2.501326	-0.056487
1	5.371174	2.501326	0.056487
1	8.750255	-0.401401	-1.716755
1	10.766482	-1.751292	-1.879343
7	11.428840	-3.860756	-0.304725
1	9.324709	-4.239740	1.366889
1	7.316110	-2.866963	1.497976
1	7.316110	2.866963	-1.497976
1	9.324709	4.239740	-1.366889
7	11.428840	3.860756	0.304725
1	10.766482	1.751292	1.879343
1	8.750255	0.401401	1.716755
6	-3.677187	-12.265843	-1.535904
6	-5.137766	-11.452970	0.274905
6	5.137766	-11.452970	-0.274905
6	3.677187	-12.265843	1.535904
6	12.286479	-3.715737	-1.466178
6	11.454599	-5.146648	0.367968
6	12.286479	3.715737	1.466178
6	11.454599	5.146648	-0.367968
6	5.137766	11.452970	0.274905
6	3.677187	12.265843	-1.535904
6	-3.677187	12.265843	1.535904
6	-5.137766	11.452970	-0.274905
6	-11.454599	5.146648	0.367968
6	-12.286479	3.715737	-1.466178
6	-12.286479	-3.715737	1.466178

6	-11.454599	-5.146648	-0.367968
1	-2.638758	-12.622228	-1.629443
1	-3.941331	-11.750005	-2.483976
1	-4.318338	-13.153115	-1.435433
1	-5.593586	-12.442388	0.127895
1	-5.835338	-10.684226	-0.121080
1	-5.049367	-11.292859	1.361627
1	5.835338	-10.684226	0.121080
1	5.049367	-11.292859	-1.361627
1	5.593586	-12.442388	-0.127895
1	4.318338	-13.153115	1.435433
1	2.638758	-12.622228	1.629443
1	3.941331	-11.750005	2.483976
1	12.653686	-2.680832	-1.558756
1	11.782384	-3.980061	-2.419783
1	13.165725	-4.364525	-1.349278
1	10.682670	-5.851779	-0.005766
1	11.300527	-5.028281	1.452797
1	12.441901	-5.608283	0.227626
1	12.653686	2.680832	1.558756
1	11.782384	3.980061	2.419783
1	13.165725	4.364525	1.349278
1	10.682670	5.851779	0.005766
1	11.300527	5.028281	-1.452797
1	12.441901	5.608283	-0.227626
1	5.835338	10.684226	-0.121080
1	5.049367	11.292859	1.361627
1	5.593586	12.442388	0.127895
1	2.638758	12.622228	-1.629443
1	3.941331	11.750005	-2.483976
1	4.318338	13.153115	-1.435433
1	-2.638758	12.622228	1.629443
1	-3.941331	11.750005	2.483976
1	-4.318338	13.153115	1.435433
1	-5.835338	10.684226	0.121080
1	-5.049367	11.292859	-1.361627
1	-5.593586	12.442388	-0.127895
1	-10.682670	5.851779	-0.005766
1	-11.300527	5.028281	1.452797
1	-12.441901	5.608283	0.227626
1	-12.653686	2.680832	-1.558756
1	-11.782384	3.980061	-2.419783
1	-13.165725	4.364525	-1.349278
1	-12.653686	-2.680832	1.558756
1	-11.782384	-3.980061	2.419783
1	-13.165725	-4.364525	1.349278
1	-10.682670	-5.851779	0.005766
1	-11.300527	-5.028281	-1.452797
1	-12.441901	-5.608283	-0.227626
47	0.000000	0.000000	0.000000

Table S11. Atom coordinates of [3][dmaphPc]- in D2 symmetry (in Å).

6	1.137665	-2.831412	-0.000338
7	2.429081	-2.406570	-0.000902
7	0.000000	-2.066136	0.000000
6	-1.137665	-2.831412	0.000338
7	-2.429081	-2.406570	0.000902
6	-2.824385	-1.144544	0.000859
7	-2.059683	-0.000000	0.000000
6	-2.824385	1.144544	-0.000859
6	0.713866	-4.215034	-0.001509
6	-0.713866	-4.215034	0.001509
6	-4.230985	-0.706635	-0.000511
6	-4.230985	0.706635	0.000511
7	-2.429081	2.406570	-0.000902
6	-1.137665	2.831412	-0.000338
7	-0.000000	2.066136	0.000000
6	1.137665	2.831412	0.000338
6	-0.713866	4.215034	-0.001509
6	0.713866	4.215034	0.001509
7	2.429081	2.406570	0.000902
6	2.824385	1.144544	0.000859
7	2.059683	0.000000	0.000000
6	2.824385	-1.144544	-0.000859
6	4.230985	0.706635	-0.000511
6	4.230985	-0.706635	0.000511
6	1.414102	-5.425791	0.008577
6	0.720582	-6.642113	0.013370
6	-0.720582	-6.642113	-0.013370
6	-1.414102	-5.425791	-0.008577
6	1.514567	-7.899144	0.093696
6	2.620610	-8.115328	-0.744327
6	3.399763	-9.269861	-0.663488
6	3.099003	-10.288640	0.267615
6	1.999460	-10.064332	1.128456
6	1.232718	-8.906026	1.033302
6	-1.514567	-7.899144	-0.093696
6	-2.620610	-8.115328	0.744327
6	-3.399763	-9.269861	0.663488
6	-3.099003	-10.288640	-0.267615
6	-1.999460	-10.064332	-1.128456
6	-1.232718	-8.906026	-1.033302
6	-5.429966	-1.410871	0.009713
6	-6.653745	-0.715012	0.013612
6	-6.653745	0.715012	-0.013612
6	-5.429966	1.410871	-0.009713
6	-7.908706	-1.513824	0.085598
6	-8.117475	-2.612950	-0.762627
6	-9.270282	-3.394908	-0.692354
6	-10.291191	-3.105981	0.240934
6	-10.071485	-2.014367	1.113760

6	-8.915998	-1.242934	1.027118
6	-7.908706	1.513824	-0.085598
6	-8.915998	1.242934	-1.027118
6	-10.071485	2.014367	-1.113760
6	-10.291191	3.105981	-0.240934
6	-9.270282	3.394908	0.692354
6	-8.117475	2.612950	0.762627
6	-1.414102	5.425791	0.008577
6	-0.720582	6.642113	0.013370
6	0.720582	6.642113	-0.013370
6	1.414102	5.425791	-0.008577
6	-1.514567	7.899144	0.093696
6	-2.620610	8.115328	-0.744327
6	-3.399763	9.269861	-0.663488
6	-3.099003	10.288640	0.267615
6	-1.999460	10.064332	1.128456
6	-1.232718	8.906026	1.033302
6	1.514567	7.899144	-0.093696
6	2.620610	8.115328	0.744327
6	3.399763	9.269861	0.663488
6	3.099003	10.288640	-0.267615
6	1.999460	10.064332	-1.128456
6	1.232718	8.906026	-1.033302
6	5.429966	1.410871	0.009713
6	6.653745	0.715012	0.013612
6	6.653745	-0.715012	-0.013612
6	5.429966	-1.410871	-0.009713
6	7.908706	1.513824	0.085598
6	8.915998	1.242934	1.027118
6	10.071485	2.014367	1.113760
6	10.291191	3.105981	0.240934
6	9.270282	3.394908	-0.692354
6	8.117475	2.612950	-0.762627
6	7.908706	-1.513824	-0.085598
6	8.117475	-2.612950	0.762627
6	9.270282	-3.394908	0.692354
6	10.291191	-3.105981	-0.240934
6	10.071485	-2.014367	-1.113760
6	8.915998	-1.242934	-1.027118
1	2.504930	-5.413748	0.051606
1	-2.504930	-5.413748	-0.051606
1	2.875600	-7.358835	-1.490234
1	4.243554	-9.370918	-1.344911
7	3.847961	-11.474062	0.327017
1	1.730259	-10.797628	1.887493
1	0.391026	-8.775622	1.715351
1	-2.875600	-7.358835	1.490234
1	-4.243554	-9.370918	1.344911
7	-3.847961	-11.474062	-0.327017
1	-1.730259	-10.797628	-1.887493
1	-0.391026	-8.775622	-1.715351

1	-5.413587	-2.501461	0.049348
1	-5.413587	2.501461	-0.049348
1	-7.357975	-2.860566	-1.507951
1	-9.367530	-4.232972	-1.381102
7	-11.471162	-3.859094	0.292697
1	-10.806102	-1.756067	1.875127
1	-8.788638	-0.407181	1.716987
1	-8.788638	0.407181	-1.716987
1	-10.806102	1.756067	-1.875127
7	-11.471162	3.859094	-0.292697
1	-9.367530	4.232972	1.381102
1	-7.357975	2.860566	1.507951
1	-2.504930	5.413748	0.051606
1	2.504930	5.413748	-0.051606
1	-2.875600	7.358835	-1.490234
1	-4.243554	9.370918	-1.344911
7	-3.847961	11.474062	0.327017
1	-1.730259	10.797628	1.887493
1	-0.391026	8.775622	1.715351
1	2.875600	7.358835	1.490234
1	4.243554	9.370918	1.344911
7	3.847961	11.474062	-0.327017
1	1.730259	10.797628	-1.887493
1	0.391026	8.775622	-1.715351
1	5.413587	2.501461	0.049348
1	5.413587	-2.501461	-0.049348
1	8.788638	0.407181	1.716987
1	10.806102	1.756067	1.875127
7	11.471162	3.859094	0.292697
1	9.367530	4.232972	-1.381102
1	7.357975	2.860566	-1.507951
1	7.357975	-2.860566	1.507951
1	9.367530	-4.232972	1.381102
7	11.471162	-3.859094	-0.292697
1	10.806102	-1.756067	-1.875127
1	8.788638	-0.407181	-1.716987
6	-3.701069	12.318042	1.497956
6	-5.144618	11.495725	-0.324009
6	5.144618	11.495725	0.324009
6	3.701069	12.318042	-1.497956
6	12.326905	3.718746	1.455996
6	11.497000	5.143021	-0.383422
6	12.326905	-3.718746	-1.455996
6	11.497000	-5.143021	0.383422
6	5.144618	-11.495725	-0.324009
6	3.701069	-12.318042	1.497956
6	-3.701069	-12.318042	-1.497956
6	-5.144618	-11.495725	0.324009
6	-11.497000	-5.143021	-0.383422
6	-12.326905	-3.718746	1.455996
6	-12.326905	3.718746	-1.455996

6	-11.497000	5.143021	0.383422
1	-2.664375	12.678090	1.596617
1	-3.970166	11.805920	2.446464
1	-4.344238	13.202715	1.388767
1	-5.603665	12.484419	-0.182924
1	-5.844703	10.726276	0.065594
1	-5.044172	11.334629	-1.409521
1	5.844703	10.726276	-0.065594
1	5.044172	11.334629	1.409521
1	5.603665	12.484419	0.182924
1	4.344238	13.202715	-1.388767
1	2.664375	12.678090	-1.596617
1	3.970166	11.805920	-2.446464
1	12.693204	2.683996	1.553794
1	11.821725	3.987595	2.407857
1	13.206863	4.366355	1.337506
1	10.725123	5.849467	-0.011758
1	11.342804	5.021742	-1.467930
1	12.484382	5.605010	-0.244467
1	12.693204	-2.683996	-1.553794
1	11.821725	-3.987595	-2.407857
1	13.206863	-4.366355	-1.337506
1	10.725123	-5.849467	0.011758
1	11.342804	-5.021742	1.467930
1	12.484382	-5.605010	0.244467
1	5.844703	-10.726276	0.065594
1	5.044172	-11.334629	-1.409521
1	5.603665	-12.484419	-0.182924
1	2.664375	-12.678090	1.596617
1	3.970166	-11.805920	2.446464
1	4.344238	-13.202715	1.388767
1	-2.664375	-12.678090	-1.596617
1	-3.970166	-11.805920	-2.446464
1	-4.344238	-13.202715	-1.388767
1	-5.844703	-10.726276	-0.065594
1	-5.044172	-11.334629	1.409521
1	-5.603665	-12.484419	0.182924
1	-10.725123	-5.849467	-0.011758
1	-11.342804	-5.021742	-1.467930
1	-12.484382	-5.605010	-0.244467
1	-12.693204	-2.683996	1.553794
1	-11.821725	-3.987595	2.407857
1	-13.206863	-4.366355	1.337506
1	-12.693204	2.683996	-1.553794
1	-11.821725	3.987595	-2.407857
1	-13.206863	4.366355	-1.337506
1	-10.725123	5.849467	0.011758
1	-11.342804	5.021742	1.467930
1	-12.484382	5.605010	0.244467
47	-0.000000	0.000000	0.000000

Table S12. Atom coordinates of [2][dmaphPc]$^{2-}$ in D_2 symmetry (in Å).

6	1.194146	2.814218	0.000634
7	0.000000	3.431632	0.000000
7	1.463229	1.463194	0.000013
6	2.814256	1.194141	-0.000663
7	3.431686	-0.000000	0.000000
6	2.814256	-1.194141	0.000663
7	1.463229	-1.463194	-0.000013
6	1.194146	-2.814218	-0.000634
6	2.479664	3.490278	-0.001018
6	3.490293	2.479670	0.000910
6	3.490293	-2.479670	-0.000910
6	2.479664	-3.490278	0.001018
7	-0.000000	-3.431632	0.000000
6	-1.194146	-2.814218	0.000634
7	-1.463229	-1.463194	0.000013
6	-2.814256	-1.194141	-0.000663
6	-2.479664	-3.490278	-0.001018
6	-3.490293	-2.479670	0.000910
7	-3.431686	0.000000	0.000000
6	-2.814256	1.194141	0.000663
7	-1.463229	1.463194	-0.000013
6	-1.194146	2.814218	-0.000634
6	-3.490293	2.479670	-0.000910
6	-2.479664	3.490278	0.001018
6	2.841833	4.838474	0.010740
6	4.194038	5.214113	0.015858
6	5.214085	4.194085	-0.016423
6	4.838480	2.841871	-0.011104
6	4.513157	6.662329	0.122501
6	3.845164	7.618160	-0.663474
6	4.099279	8.985814	-0.551778
6	5.062520	9.477952	0.354875
6	5.723935	8.524771	1.161439
6	5.455538	7.163138	1.039939
6	6.662280	4.513233	-0.123305
6	7.618239	3.845393	0.662647
6	8.985870	4.099543	0.550735
6	9.477853	5.062675	-0.356114
6	8.524542	5.723944	-1.162641
6	7.162936	5.455518	-1.040926
6	4.838480	-2.841871	0.011104
6	5.214085	-4.194085	0.016423
6	4.194038	-5.214113	-0.015858
6	2.841833	-4.838474	-0.010740
6	6.662280	-4.513233	0.123305
6	7.618239	-3.845393	-0.662647
6	8.985870	-4.099543	-0.550735
6	9.477853	-5.062675	0.356114
6	8.524542	-5.723944	1.162641

6	7.162936	-5.455518	1.040926
6	4.513157	-6.662329	-0.122501
6	5.455538	-7.163138	-1.039939
6	5.723935	-8.524771	-1.161439
6	5.062520	-9.477952	-0.354875
6	4.099279	-8.985814	0.551778
6	3.845164	-7.618160	0.663474
6	-2.841833	-4.838474	0.010740
6	-4.194038	-5.214113	0.015858
6	-5.214085	-4.194085	-0.016423
6	-4.838480	-2.841871	-0.011104
6	-4.513157	-6.662329	0.122501
6	-3.845164	-7.618160	-0.663474
6	-4.099279	-8.985814	-0.551778
6	-5.062520	-9.477952	0.354875
6	-5.723935	-8.524771	1.161439
6	-5.455538	-7.163138	1.039939
6	-6.662280	-4.513233	-0.123305
6	-7.618239	-3.845393	0.662647
6	-8.985870	-4.099543	0.550735
6	-9.477853	-5.062675	-0.356114
6	-8.524542	-5.723944	-1.162641
6	-7.162936	-5.455518	-1.040926
6	-4.838480	2.841871	0.011104
6	-5.214085	4.194085	0.016423
6	-4.194038	5.214113	-0.015858
6	-2.841833	4.838474	-0.010740
6	-6.662280	4.513233	0.123305
6	-7.162936	5.455518	1.040926
6	-8.524542	5.723944	1.162641
6	-9.477853	5.062675	0.356114
6	-8.985870	4.099543	-0.550735
6	-7.618239	3.845393	-0.662647
6	-4.513157	6.662329	-0.122501
6	-3.845164	7.618160	0.663474
6	-4.099279	8.985814	0.551778
6	-5.062520	9.477952	-0.354875
6	-5.723935	8.524771	-1.161439
6	-5.455538	7.163138	-1.039939
1	2.057694	5.597172	0.061359
1	5.597192	2.057746	-0.061732
1	3.105051	7.278101	-1.391384
1	3.539554	9.666311	-1.192864
7	5.362873	10.854996	0.439396
1	6.459394	8.837860	1.901917
1	5.988942	6.463618	1.685398
1	7.278306	3.105373	1.390709
1	9.666471	3.539929	1.191806
7	10.854881	5.363102	-0.440837
1	8.837508	6.459318	-1.903257
1	6.463311	5.988811	-1.686361

1	5.597192	-2.057746	0.061732
1	2.057694	-5.597172	-0.061359
1	7.278306	-3.105373	-1.390709
1	9.666471	-3.539929	-1.191806
7	10.854881	-5.363102	0.440837
1	8.837508	-6.459318	1.903257
1	6.463311	-5.988811	1.686361
1	5.988942	-6.463618	-1.685398
1	6.459394	-8.837860	-1.901917
7	5.362873	-10.854996	-0.439396
1	3.539554	-9.666311	1.192864
1	3.105051	-7.278101	1.391384
1	-2.057694	-5.597172	0.061359
1	-5.597192	-2.057746	-0.061732
1	-3.105051	-7.278101	-1.391384
1	-3.539554	-9.666311	-1.192864
7	-5.362873	-10.854996	0.439396
1	-6.459394	-8.837860	1.901917
1	-5.988942	-6.463618	1.685398
1	-7.278306	-3.105373	1.390709
1	-9.666471	-3.539929	1.191806
7	-10.854881	-5.363102	-0.440837
1	-8.837508	-6.459318	-1.903257
1	-6.463311	-5.988811	-1.686361
1	-5.597192	2.057746	0.061732
1	-2.057694	5.597172	-0.061359
1	-6.463311	5.988811	1.686361
1	-8.837508	6.459318	1.903257
7	-10.854881	5.363102	0.440837
1	-9.666471	3.539929	-1.191806
1	-7.278306	3.105373	-1.390709
1	-3.105051	7.278101	1.391384
1	-3.539554	9.666311	1.192864
7	-5.362873	10.854996	-0.439396
1	-6.459394	8.837860	-1.901917
1	-5.988942	6.463618	-1.685398
6	-6.070462	-11.314837	1.618999
6	-4.391927	-11.785488	-0.105686
6	-11.785478	-4.392238	0.104221
6	-11.314544	-6.070403	-1.620691
6	-11.314544	6.070403	1.620691
6	-11.785478	4.392238	-0.104221
6	-6.070462	11.314837	-1.618999
6	-4.391927	11.785488	0.105686
6	4.391927	11.785488	-0.105686
6	6.070462	11.314837	1.618999
6	11.314544	6.070403	-1.620691
6	11.785478	4.392238	0.104221
6	11.785478	-4.392238	-0.104221
6	11.314544	-6.070403	1.620691
6	6.070462	-11.314837	-1.618999

6	4.391927	-11.785488	0.105686
1	-7.061649	-10.839853	1.692338
1	-5.529894	-11.109269	2.568934
1	-6.234030	-12.399952	1.542324
1	-4.754130	-12.813649	0.041622
1	-3.386064	-11.697995	0.359556
1	-4.269121	-11.636039	-1.190125
1	-11.698056	-3.386357	-0.361002
1	-11.636088	-4.269443	1.188667
1	-12.813602	-4.754533	-0.043134
1	-12.399651	-6.234092	-1.544164
1	-10.839464	-7.061524	-1.694257
1	-11.108935	-5.529543	-2.570450
1	-10.839464	7.061524	1.694257
1	-11.108935	5.529543	2.570450
1	-12.399651	6.234092	1.544164
1	-11.698056	3.386357	0.361002
1	-11.636088	4.269443	-1.188667
1	-12.813602	4.754533	0.043134
1	-7.061649	10.839853	-1.692338
1	-5.529894	11.109269	-2.568934
1	-6.234030	12.399952	-1.542324
1	-3.386064	11.697995	-0.359556
1	-4.269121	11.636039	1.190125
1	-4.754130	12.813649	-0.041622
1	3.386064	11.697995	0.359556
1	4.269121	11.636039	-1.190125
1	4.754130	12.813649	0.041622
1	7.061649	10.839853	1.692338
1	5.529894	11.109269	2.568934
1	6.234030	12.399952	1.542324
1	10.839464	7.061524	-1.694257
1	11.108935	5.529543	-2.570450
1	12.399651	6.234092	-1.544164
1	11.698056	3.386357	-0.361002
1	11.636088	4.269443	1.188667
1	12.813602	4.754533	-0.043134
1	11.698056	-3.386357	0.361002
1	11.636088	-4.269443	-1.188667
1	12.813602	-4.754533	0.043134
1	10.839464	-7.061524	1.694257
1	11.108935	-5.529543	2.570450
1	12.399651	-6.234092	1.544164
1	7.061649	-10.839853	-1.692338
1	5.529894	-11.109269	-2.568934
1	6.234030	-12.399952	-1.542324
1	3.386064	-11.697995	-0.359556
1	4.269121	-11.636039	1.190125
1	4.754130	-12.813649	-0.041622
47	0.000000	0.000000	0.000000

Table S13. Atom coordinates of [4][dmaphPc]$^{2-}$ in D_{4h} symmetry (in Å).

6	-1.146295	2.835333	0.000000
7	-2.427655	2.427655	-0.000000
7	0.000000	2.070034	0.000000
6	1.146295	2.835333	0.000000
7	2.427655	2.427655	0.000000
6	2.835333	1.146295	0.000000
7	2.070034	-0.000000	0.000000
6	2.835333	-1.146295	-0.000000
6	-0.715620	4.222401	0.000000
6	0.715620	4.222401	0.000000
6	4.222401	0.715620	0.000000
6	4.222401	-0.715620	-0.000000
7	2.427655	-2.427655	-0.000000
6	1.146295	-2.835333	0.000000
7	-0.000000	-2.070034	0.000000
6	-1.146295	-2.835333	-0.000000
6	0.715620	-4.222401	0.000000
6	-0.715620	-4.222401	-0.000000
7	-2.427655	-2.427655	-0.000000
6	-2.835333	-1.146295	0.000000
7	-2.070034	0.000000	0.000000
6	-2.835333	1.146295	-0.000000
6	-4.222401	-0.715620	0.000000
6	-4.222401	0.715620	-0.000000
6	-1.418191	5.433021	0.000000
6	-0.716126	6.643189	0.000000
6	0.716126	6.643189	0.000000
6	1.418191	5.433021	0.000000
6	-1.467022	7.936076	0.000000
6	-1.840947	8.569590	1.193112
6	-2.547799	9.773401	1.204216
6	-2.907605	10.416754	0.000000
6	-2.547799	9.773401	-1.204216
6	-1.840947	8.569590	-1.193112
6	1.467022	7.936076	0.000000
6	1.840947	8.569590	-1.193112
6	2.547799	9.773401	-1.204216
6	2.907605	10.416754	0.000000
6	2.547799	9.773401	1.204216
6	1.840947	8.569590	1.193112
6	5.433021	1.418191	0.000000
6	6.643189	0.716126	0.000000
6	6.643189	-0.716126	-0.000000
6	5.433021	-1.418191	-0.000000
6	7.936076	1.467022	0.000000
6	8.569590	1.840947	1.193112
6	9.773401	2.547799	1.204216
6	10.416754	2.907605	0.000000
6	9.773401	2.547799	-1.204216

6	8.569590	1.840947	-1.193112
6	7.936076	-1.467022	-0.000000
6	8.569590	-1.840947	1.193112
6	9.773401	-2.547799	1.204216
6	10.416754	-2.907605	-0.000000
6	9.773401	-2.547799	-1.204216
6	8.569590	-1.840947	-1.193112
6	1.418191	-5.433021	0.000000
6	0.716126	-6.643189	0.000000
6	-0.716126	-6.643189	-0.000000
6	-1.418191	-5.433021	-0.000000
6	1.467022	-7.936076	0.000000
6	1.840947	-8.569590	1.193112
6	2.547799	-9.773401	1.204216
6	2.907605	-10.416754	0.000000
6	2.547799	-9.773401	-1.204216
6	1.840947	-8.569590	-1.193112
6	-1.467022	-7.936076	-0.000000
6	-1.840947	-8.569590	-1.193112
6	-2.547799	-9.773401	-1.204216
6	-2.907605	-10.416754	-0.000000
6	-2.547799	-9.773401	1.204216
6	-1.840947	-8.569590	1.193112
6	-5.433021	-1.418191	0.000000
6	-6.643189	-0.716126	0.000000
6	-6.643189	0.716126	-0.000000
6	-5.433021	1.418191	-0.000000
6	-7.936076	-1.467022	0.000000
6	-8.569590	-1.840947	-1.193112
6	-9.773401	-2.547799	-1.204216
6	-10.416754	-2.907605	0.000000
6	-9.773401	-2.547799	1.204216
6	-8.569590	-1.840947	1.193112
6	-7.936076	1.467022	-0.000000
6	-8.569590	1.840947	-1.193112
6	-9.773401	2.547799	-1.204216
6	-10.416754	2.907605	-0.000000
6	-9.773401	2.547799	1.204216
6	-8.569590	1.840947	1.193112
1	-2.510618	5.424362	0.000000
1	2.510618	5.424362	0.000000
1	-1.574635	8.099652	2.142704
1	-2.814985	10.207097	2.167427
7	-3.584670	11.654305	0.000000
1	-2.814985	10.207097	-2.167427
1	-1.574635	8.099652	-2.142704
1	1.574635	8.099652	-2.142704
1	2.814985	10.207097	-2.167427
7	3.584670	11.654305	0.000000
1	2.814985	10.207097	2.167427
1	1.574635	8.099652	2.142704

1	5.424362	2.510618	0.000000
1	5.424362	-2.510618	-0.000000
1	8.099652	1.574635	2.142704
1	10.207097	2.814985	2.167427
7	11.654305	3.584670	0.000000
1	10.207097	2.814985	-2.167427
1	8.099652	1.574635	-2.142704
1	8.099652	-1.574635	2.142704
1	10.207097	-2.814985	2.167427
7	11.654305	-3.584670	-0.000000
1	10.207097	-2.814985	-2.167427
1	8.099652	-1.574635	-2.142704
1	2.510618	-5.424362	0.000000
1	-2.510618	-5.424362	-0.000000
1	1.574635	-8.099652	2.142704
1	2.814985	-10.207097	2.167427
7	3.584670	-11.654305	0.000000
1	2.814985	-10.207097	-2.167427
1	1.574635	-8.099652	-2.142704
1	-1.574635	-8.099652	-2.142704
1	-2.814985	-10.207097	-2.167427
7	-3.584670	-11.654305	-0.000000
1	-2.814985	-10.207097	2.167427
1	-1.574635	-8.099652	2.142704
1	-5.424362	-2.510618	0.000000
1	-5.424362	2.510618	-0.000000
1	-8.099652	-1.574635	-2.142704
1	-10.207097	-2.814985	-2.167427
7	-11.654305	-3.584670	0.000000
1	-10.207097	-2.814985	2.167427
1	-8.099652	-1.574635	2.142704
1	-8.099652	1.574635	-2.142704
1	-10.207097	2.814985	-2.167427
7	-11.654305	3.584670	-0.000000
1	-10.207097	2.814985	2.167427
1	-8.099652	1.574635	2.142704
6	4.241081	-12.061649	-1.227539
6	4.241081	-12.061649	1.227539
6	-4.241081	-12.061649	-1.227539
6	-4.241081	-12.061649	1.227539
6	-12.061649	-4.241081	-1.227539
6	-12.061649	-4.241081	1.227539
6	-12.061649	4.241081	1.227539
6	-12.061649	4.241081	-1.227539
6	-4.241081	12.061649	1.227539
6	-4.241081	12.061649	-1.227539
6	4.241081	12.061649	1.227539
6	4.241081	12.061649	-1.227539
6	12.061649	4.241081	1.227539
6	12.061649	4.241081	-1.227539
6	12.061649	-4.241081	1.227539

6	12.061649	-4.241081	-1.227539
1	3.509405	-12.189411	-2.041259
1	5.009873	-11.339724	-1.579169
1	4.727061	-13.035963	-1.071470
1	4.727061	-13.035963	1.071470
1	5.009873	-11.339724	1.579169
1	3.509405	-12.189411	2.041259
1	-5.009873	-11.339724	-1.579169
1	-3.509405	-12.189411	-2.041259
1	-4.727061	-13.035963	-1.071470
1	-4.727061	-13.035963	1.071470
1	-3.509405	-12.189411	2.041259
1	-5.009873	-11.339724	1.579169
1	-12.189411	-3.509405	-2.041259
1	-11.339724	-5.009873	-1.579169
1	-13.035963	-4.727061	-1.071470
1	-11.339724	-5.009873	1.579169
1	-12.189411	-3.509405	2.041259
1	-13.035963	-4.727061	1.071470
1	-12.189411	3.509405	2.041259
1	-11.339724	5.009873	1.579169
1	-13.035963	4.727061	1.071470
1	-11.339724	5.009873	-1.579169
1	-12.189411	3.509405	-2.041259
1	-13.035963	4.727061	-1.071470
1	-5.009873	11.339724	1.579169
1	-3.509405	12.189411	2.041259
1	-4.727061	13.035963	1.071470
1	-3.509405	12.189411	-2.041259
1	-5.009873	11.339724	-1.579169
1	-4.727061	13.035963	-1.071470
1	3.509405	12.189411	2.041259
1	5.009873	11.339724	1.579169
1	4.727061	13.035963	1.071470
1	5.009873	11.339724	-1.579169
1	3.509405	12.189411	-2.041259
1	4.727061	13.035963	-1.071470
1	11.339724	5.009873	1.579169
1	12.189411	3.509405	2.041259
1	13.035963	4.727061	1.071470
1	12.189411	3.509405	-2.041259
1	11.339724	5.009873	-1.579169
1	13.035963	4.727061	-1.071470
1	12.189411	-3.509405	2.041259
1	11.339724	-5.009873	1.579169
1	13.035963	-4.727061	1.071470
1	11.339724	-5.009873	-1.579169
1	12.189411	-3.509405	-2.041259
1	13.035963	-4.727061	-1.071470
47	0.000000	0.000000	0.000000

Table S14. Atom coordinates of [4][dmaphPc]$^{2-}$ in D_4 symmetry (in Å).

6	-1.145782	2.834374	0.000549
7	-2.426257	2.426257	0.000000
7	0.000000	2.068806	0.000000
6	1.145782	2.834374	-0.000549
7	2.426257	2.426257	0.000000
6	2.834374	1.145782	0.000549
7	2.068806	-0.000000	0.000000
6	2.834374	-1.145782	-0.000549
6	-0.714616	4.221334	-0.001138
6	0.714616	4.221334	0.001138
6	4.221334	0.714616	-0.001138
6	4.221334	-0.714616	0.001138
7	2.426257	-2.426257	0.000000
6	1.145782	-2.834374	0.000549
7	-0.000000	-2.068806	0.000000
6	-1.145782	-2.834374	-0.000549
6	0.714616	-4.221334	-0.001138
6	-0.714616	-4.221334	0.001138
7	-2.426257	-2.426257	-0.000000
6	-2.834374	-1.145782	0.000549
7	-2.068806	0.000000	0.000000
6	-2.834374	1.145782	-0.000549
6	-4.221334	-0.714616	-0.001138
6	-4.221334	0.714616	0.001138
6	-1.411763	5.430861	0.010143
6	-0.721154	6.652623	0.015461
6	0.721154	6.652623	-0.015461
6	1.411763	5.430861	-0.010143
6	-1.519618	7.902526	0.120335
6	-2.665556	8.106565	-0.668732
6	-3.452780	9.253657	-0.558976
6	-3.121840	10.282320	0.348956
6	-1.982446	10.075448	1.158611
6	-1.209508	8.922596	1.038891
6	1.519618	7.902526	-0.120335
6	2.665556	8.106565	0.668732
6	3.452780	9.253657	0.558976
6	3.121840	10.282320	-0.348956
6	1.982446	10.075448	-1.158611
6	1.209508	8.922596	-1.038891
6	5.430861	1.411763	0.010143
6	6.652623	0.721154	0.015461
6	6.652623	-0.721154	-0.015461
6	5.430861	-1.411763	-0.010143
6	7.902526	1.519618	0.120335
6	8.106565	2.665556	-0.668732
6	9.253657	3.452780	-0.558976
6	10.282320	3.121840	0.348956
6	10.075448	1.982446	1.158611

6	8.922596	1.209508	1.038891
6	7.902526	-1.519618	-0.120335
6	8.922596	-1.209508	-1.038891
6	10.075448	-1.982446	-1.158611
6	10.282320	-3.121840	-0.348956
6	9.253657	-3.452780	0.558976
6	8.106565	-2.665556	0.668732
6	1.411763	-5.430861	0.010143
6	0.721154	-6.652623	0.015461
6	-0.721154	-6.652623	-0.015461
6	-1.411763	-5.430861	-0.010143
6	1.519618	-7.902526	0.120335
6	2.665556	-8.106565	-0.668732
6	3.452780	-9.253657	-0.558976
6	3.121840	-10.282320	0.348956
6	1.982446	-10.075448	1.158611
6	1.209508	-8.922596	1.038891
6	-1.519618	-7.902526	-0.120335
6	-2.665556	-8.106565	0.668732
6	-3.452780	-9.253657	0.558976
6	-3.121840	-10.282320	-0.348956
6	-1.982446	-10.075448	-1.158611
6	-1.209508	-8.922596	-1.038891
6	-5.430861	-1.411763	0.010143
6	-6.652623	-0.721154	0.015461
6	-6.652623	0.721154	-0.015461
6	-5.430861	1.411763	-0.010143
6	-7.902526	-1.519618	0.120335
6	-8.922596	-1.209508	1.038891
6	-10.075448	-1.982446	1.158611
6	-10.282320	-3.121840	0.348956
6	-9.253657	-3.452780	-0.558976
6	-8.106565	-2.665556	-0.668732
6	-7.902526	1.519618	-0.120335
6	-8.106565	2.665556	0.668732
6	-9.253657	3.452780	0.558976
6	-10.282320	3.121840	-0.348956
6	-10.075448	1.982446	-1.158611
6	-8.922596	1.209508	-1.038891
1	-2.502757	5.413078	0.060015
1	2.502757	5.413078	-0.060015
1	-2.946670	7.342902	-1.397470
1	-4.327914	9.339611	-1.202486
7	-3.883070	11.468612	0.431902
1	-1.685504	10.816619	1.900067
1	-0.339436	8.804553	1.686582
1	2.946670	7.342902	1.397470
1	4.327914	9.339611	1.202486
7	3.883070	11.468612	-0.431902
1	1.685504	10.816619	-1.900067
1	0.339436	8.804553	-1.686582

1	5.413078	2.502757	0.060015
1	5.413078	-2.502757	-0.060015
1	7.342902	2.946670	-1.397470
1	9.339611	4.327914	-1.202486
7	11.468612	3.883070	0.431902
1	10.816619	1.685504	1.900067
1	8.804553	0.339436	1.686582
1	8.804553	-0.339436	-1.686582
1	10.816619	-1.685504	-1.900067
7	11.468612	-3.883070	-0.431902
1	9.339611	-4.327914	1.202486
1	7.342902	-2.946670	1.397470
1	2.502757	-5.413078	0.060015
1	-2.502757	-5.413078	-0.060015
1	2.946670	-7.342902	-1.397470
1	4.327914	-9.339611	-1.202486
7	3.883070	-11.468612	0.431902
1	1.685504	-10.816619	1.900067
1	0.339436	-8.804553	1.686582
1	-2.946670	-7.342902	1.397470
1	-4.327914	-9.339611	1.202486
7	-3.883070	-11.468612	-0.431902
1	-1.685504	-10.816619	-1.900067
1	-0.339436	-8.804553	-1.686582
1	-5.413078	-2.502757	0.060015
1	-5.413078	2.502757	-0.060015
1	-8.804553	-0.339436	1.686582
1	-10.816619	-1.685504	1.900067
7	-11.468612	-3.883070	0.431902
1	-9.339611	-4.327914	-1.202486
1	-7.342902	-2.946670	-1.397470
1	-7.342902	2.946670	1.397470
1	-9.339611	4.327914	1.202486
7	-11.468612	3.883070	-0.431902
1	-10.816619	1.685504	-1.900067
1	-8.804553	0.339436	-1.686582
6	3.711465	-12.292974	1.612843
6	5.226034	-11.440659	-0.117128
6	-5.226034	-11.440659	0.117128
6	-3.711465	-12.292974	-1.612843
6	-12.292974	-3.711465	1.612843
6	-11.440659	-5.226034	-0.117128
6	-12.292974	3.711465	-1.612843
6	-11.440659	5.226034	0.117128
6	-5.226034	11.440659	-0.117128
6	-3.711465	12.292974	1.612843
6	3.711465	12.292974	-1.612843
6	5.226034	11.440659	0.117128
6	11.440659	5.226034	-0.117128
6	12.292974	3.711465	1.612843
6	12.292974	-3.711465	-1.612843

6	11.440659	-5.226034	0.117128
1	2.674904	-12.657732	1.689862
1	3.951485	-11.764520	2.561504
1	4.362650	-13.176144	1.534926
1	5.697171	-12.423769	0.029593
1	5.876856	-10.667281	0.345665
1	5.204072	-11.248941	-1.201657
1	-5.876856	-10.667281	-0.345665
1	-5.204072	-11.248941	1.201657
1	-5.697171	-12.423769	-0.029593
1	-4.362650	-13.176144	-1.534926
1	-2.674904	-12.657732	-1.689862
1	-3.951485	-11.764520	-2.561504
1	-12.657732	-2.674904	1.689862
1	-11.764520	-3.951485	2.561504
1	-13.176144	-4.362650	1.534926
1	-10.667281	-5.876856	0.345665
1	-11.248941	-5.204072	-1.201657
1	-12.423769	-5.697171	0.029593
1	-12.657732	2.674904	-1.689862
1	-11.764520	3.951485	-2.561504
1	-13.176144	4.362650	-1.534926
1	-10.667281	5.876856	-0.345665
1	-11.248941	5.204072	1.201657
1	-12.423769	5.697171	-0.029593
1	-5.876856	10.667281	0.345665
1	-5.204072	11.248941	-1.201657
1	-5.697171	12.423769	0.029593
1	-2.674904	12.657732	1.689862
1	-3.951485	11.764520	2.561504
1	-4.362650	13.176144	1.534926
1	2.674904	12.657732	-1.689862
1	3.951485	11.764520	-2.561504
1	4.362650	13.176144	-1.534926
1	5.876856	10.667281	-0.345665
1	5.204072	11.248941	1.201657
1	5.697171	12.423769	-0.029593
1	10.667281	5.876856	0.345665
1	11.248941	5.204072	-1.201657
1	12.423769	5.697171	0.029593
1	12.657732	2.674904	1.689862
1	11.764520	3.951485	2.561504
1	13.176144	4.362650	1.534926
1	12.657732	-2.674904	-1.689862
1	11.764520	-3.951485	-2.561504
1	13.176144	-4.362650	-1.534926
1	10.667281	-5.876856	-0.345665
1	11.248941	-5.204072	1.201657
1	12.423769	-5.697171	-0.029593
47	0.000000	0.000000	0.000000
